# IL-6 signaling orchestrates proteolytic hubs MuRF1 and Atrogin-1 in NSCLC induced sarcopenia

**DOI:** 10.3389/fimmu.2026.1885023

**Published:** 2026-08-03

**Authors:** Gautam Kumar, Shailza Singh

**Affiliations:** 1 Systems Medicine Laboratory, BRIC- National Centre for Cell Science, NCCS Complex, Ganeshkhind, Pune, India; 2 Regional Centre for Biotechnology, Faridabad, Haryana, India

**Keywords:** Atrogin-1, IL-6, MuRF1, NSCLC, sarcopenia, signaling, therapeutics

## Abstract

Non-small cell lung cancer (NSCLC) is frequently associated with sarcopenia, a debilitating condition of muscle wasting driven by complex tumor–muscle cross-talk. To unravel the regulatory mechanisms underlying this phenotype, we reconstructed a comprehensive signaling network integrating inflammatory, anabolic, catabolic, and proteolytic pathways. The network was translated into a mechanistic mathematical model using ordinary differential equations, enabling dynamic simulations of pathway activity. Flux analysis revealed that only a limited number of reactions dominate system behavior, with cytoplasmic IL-6 export and SMAD2/3–4 mediated induction of MuRF1 and Atrogin-1 emerging as major control points for muscle protein breakdown. Crosstalk analysis identified these proteolytic regulators as central hubs, integrating signals from inflammatory cytokines, oxidative stress, and transcriptional modulators. Principal component analysis further confirmed that sarcopenic progression is governed by a compact regulatory core, with IL-6/STAT3, myostatin/SMAD, and FOXO/NF-κB pathways converging on MuRF1 and Atrogin-1. Experimental validation using immunofluorescence-based confocal microscopy demonstrated increased expression and altered localization of these ubiquitin ligases in C2C12 cells co-cultured with lung cancer lines, corroborating model predictions. Together, these findings provide a systems-level framework that transforms broad observations of inflammation into ranked therapeutic targets and support combined strategies aimed at blocking the IL-6/STAT3–myostatin/SMAD–FOXO1/3–MuRF1/Atrogin-1 axis to mitigate NSCLC-associated sarcopenia.

## Introduction

Lung cancer remains the leading cause of cancer-related mortality worldwide, accounting for approximately 1.8 million deaths annually and surpassing the combined mortality of breast, prostate, and colorectal cancers (1). Among its subtypes, non-small cell lung cancer (NSCLC) accounts for approximately 85% of all lung cancer cases and is frequently diagnosed at advanced stages (2). Beyond tumor burden itself, growing evidence has identified skeletal muscle wasting, or sarcopenia, as a major determinant of clinical outcome in NSCLC (3). Cross-sectional imaging-based assessments reveal that 40–52% of NSCLC patients present with sarcopenia at diagnosis (4). Sarcopenia is not merely a consequence of reduced physical activity or malnutrition but an independent prognostic factor associated with increased chemotherapy toxicity, impaired treatment tolerance, postoperative complications, and markedly reduced overall survival (5, 6). In lung cancer patients, the coexistence of sarcopenia serves as a potent predictor of poor survival outcomes, driven by tumor-mediated metabolic reprogramming and the competitive consumption of vital nutrients that induce a systemic catabolic state. By actively disrupting the balance between protein synthesis and degradation, the malignancy significantly accelerates progressive muscle wasting (7).

Sarcopenia is a progressive and generalized skeletal muscle disorder characterized by the loss of muscle mass, strength and physical performance, resulting in increased risk of disability, morbidity and mortality. The primary diagnostic criterion for sarcopenia, according to the European Working Group on Sarcopenia in Older People (EWGSOP2), is low muscle strength (grip strength<27 kg for men/<16 kg for women, chair stand >15 s). This is confirmed by low muscle quantity or quality (appendicular skeletal muscle mass<20 kg men/<15 kg women via DXA/BIA), and it is further classified as severe when combined with poor physical performance (gait speed<0.8 m/s; SPPB<4; TUG >20 s) (8). Clinically, sarcopenia is classified into three major types primary sarcopenia, driven predominantly by aging-related neuromuscular degeneration, secondary sarcopenia arising from chronic disease, inflammation, malnutrition or physical inactivity and acute sarcopenia, characterized by rapid muscle loss following hospitalization, surgery or critical illness (9–11). Cancer-associated sarcopenia represents a particularly aggressive subtype of secondary sarcopenia, distinguished by systemic inflammation, endocrine dysregulation and resistance to conventional nutritional or exercise interventions (12). The prevalence of sarcopenia varies widely depending on population, diagnostic criteria and disease stage (13). In NSCLC, reported prevalence ranges from 40% to over 50%, with rates highest in patients with metastatic cancer or undergoing cytotoxic chemotherapy (14). Diagnosis of sarcopenia in cancer patients relies on assessment of both muscle function and muscle mass. Muscle strength is most commonly evaluated using handgrip dynamometry or chair-stand tests, while muscle quantity is quantified using computed tomography–derived cross-sectional area of skeletal muscle at the L3 vertebral level, dual-energy X-ray absorptiometry (DXA) or bioelectrical impedance analysis, (CT L3-SMI<52 cm²/m² men,<38 women preferred in oncology) (15, 16). In NSCLC, routine staging CT scans offer a reliable and reproducible method for skeletal muscle quantification, facilitating early detection of sarcopenia without requiring additional imaging burdens.

Sarcopenia is a multifactorial syndrome characterized by a progressive disruption of skeletal muscle homeostasis. While primary sarcopenia is typically driven by aging, physical inactivity, and inadequate nutrition, cancer-associated sarcopenia represents a distinct, highly severe phenotype that progresses rapidly due to chronic systemic inflammation and tumor-derived signaling factors (17). Advanced age is the principal risk factor for primary sarcopenia, with individuals over 80 years exhibiting the highest prevalence due to cellular senescence, satellite cell exhaustion, and impaired neuromuscular regeneration (18). Female sex is also associated with an increased risk, partly owing to lower baseline muscle mass and reduced anabolic hormonal support (19). Phenotypic indicators such as a low body mass index (BMI<18.5 kg/m²) and a reduced calf circumference (<31 cm) are strongly linked to this disease, reflecting reduced protein synthesis mediated by the IGF-1/PI3K/AKT signaling pathway (20, 21). Furthermore, lifestyle behaviors like physical inactivity and smoking accelerate muscle wasting by inducing disuse atrophy, oxidative stress, and mitochondrial dysfunction (20, 22). Finally, malnutrition stands as a major modifiable contributor; specifically, deficiencies in leucine and vitamin D impair mechanistic target of rapamycin (mTORC1) signaling and suppress muscle protein synthesis, thereby exacerbating the sarcopenic phenotype (23).

Although several comorbidities, including diabetes, cardiovascular disease, osteoporosis, and endocrine dysfunction, contribute to muscle loss, chronic inflammation has emerged as the central driver of sarcopenia across aging, cancer, and other systemic diseases (24–28). This persistent low-grade inflammatory state, termed “inflammaging.” Inflammaging is characterized by sustained elevations of circulating pro-inflammatory mediators, including IL-6, TNF-α, and C-reactive protein, which disrupt the balance between protein synthesis and degradation in skeletal muscle. These cytokines activate key catabolic pathways such as JAK/STAT3 and NF-κB signaling, promoting forkhead box1/3 (FOXO1/3) nuclear translocation and transcriptional upregulation of the E3 ubiquitin ligases muscle RING-finger protein-1 (MuRF1) and Atrogin-1, the principal mediators of muscle protein breakdown (29). In parallel, inflammatory signaling impairs mitochondrial function, increases reactive oxygen species production, suppresses Akt/mTOR signaling, and reduces anabolic protein synthesis, thereby accelerating muscle loss (30, 31).

The role of inflammation is particularly pronounced in cancer-associated sarcopenia, where tumor-derived cytokines amplify systemic inflammatory responses. Among these, IL-6 is a key mediator, especially in NSCLC, where elevated IL-6 levels cooperate with TNF-α/NF-κB and myostatin/SMAD signaling to establish a dominant catabolic environment that promotes progressive muscle loss (14, 32). Consequently, sarcopenia is highly prevalent in advanced lung cancer and is strongly associated with poor clinical outcomes and reduced survival (33, 34). These observations highlight inflammatory signaling as a major molecular link between cancer progression and skeletal muscle degeneration, providing the rationale for focusing in the present study.

Despite the profound clinical impact of sarcopenia in NSCLC, there is currently no approved, disease-modifying therapy specifically targeting cancer-associated muscle wasting. Management strategies remain largely supportive and are adapted from geriatric sarcopenia, underscoring a major therapeutic gap need to consider. Nutritional supplementation and resistance exercise represent the cornerstone of sarcopenia management in older adults (35). High-protein diets enriched in leucine or β-hydroxy-β-methylbutyrate (HMB) modestly enhance muscle protein synthesis via mTORC1 activation, while supervised resistance training stimulates myofiber hypertrophy (36, 37). However, in NSCLC patients, these approaches yield only marginal benefits, reflecting profound anabolic resistance driven by inflammatory and endocrine dysregulation. Clinical trials report that nutritional intervention alone improves lean body mass by less than 5%, with minimal impact on functional outcomes during chemotherapy. Given the central role of chronic inflammation, cytokine blockade has emerged as a rational therapeutic strategy. IL-6 pathway inhibitors, including the monoclonal antibody tocilizumab and the IL-6 ligand trap siltuximab, have demonstrated improvements in muscle strength and fatigue in inflammatory disease contexts (38, 39). Earlier studies also suggest that IL-6 inhibition reduces CRP levels and stabilizes body weight, although robust sarcopenia endpoints remain underexplored. Similarly, TNF-α inhibitors attenuate NF-κB-driven catabolism in preclinical cancer cachexia models but have shown limited efficacy in advanced malignancy (40). The myostatin–SMAD2/3 axis represents one of the most promising molecular targets in cancer-associated sarcopenia. Neutralizing antibodies against myostatin and soluble ActRIIB decoy receptors have produced robust increases in muscle mass in preclinical tumor models and early clinical trials (41). However, translation has been hampered by off-target effects and lack of durable functional improvement, suggesting that myostatin blockade alone cannot override the inflammatory milieu characteristic of NSCLC. Small-molecule inhibitors of the ubiquitin–proteasome system, autophagy modulators, and agents stabilizing mitochondrial dynamics are under active investigation. Suppression of FOXO1/3 or MuRF1/Atrogin-1 transcription has shown potent anti-atrophic effects in murine cancer cachexia models (42, 43). Similarly, pharmacological inhibition of dynamin-related protein (DRP1) mediated mitochondrial fission restores oxidative capacity and rescues muscle fiber integrity under inflammatory stress (44).

**Figure 1:**
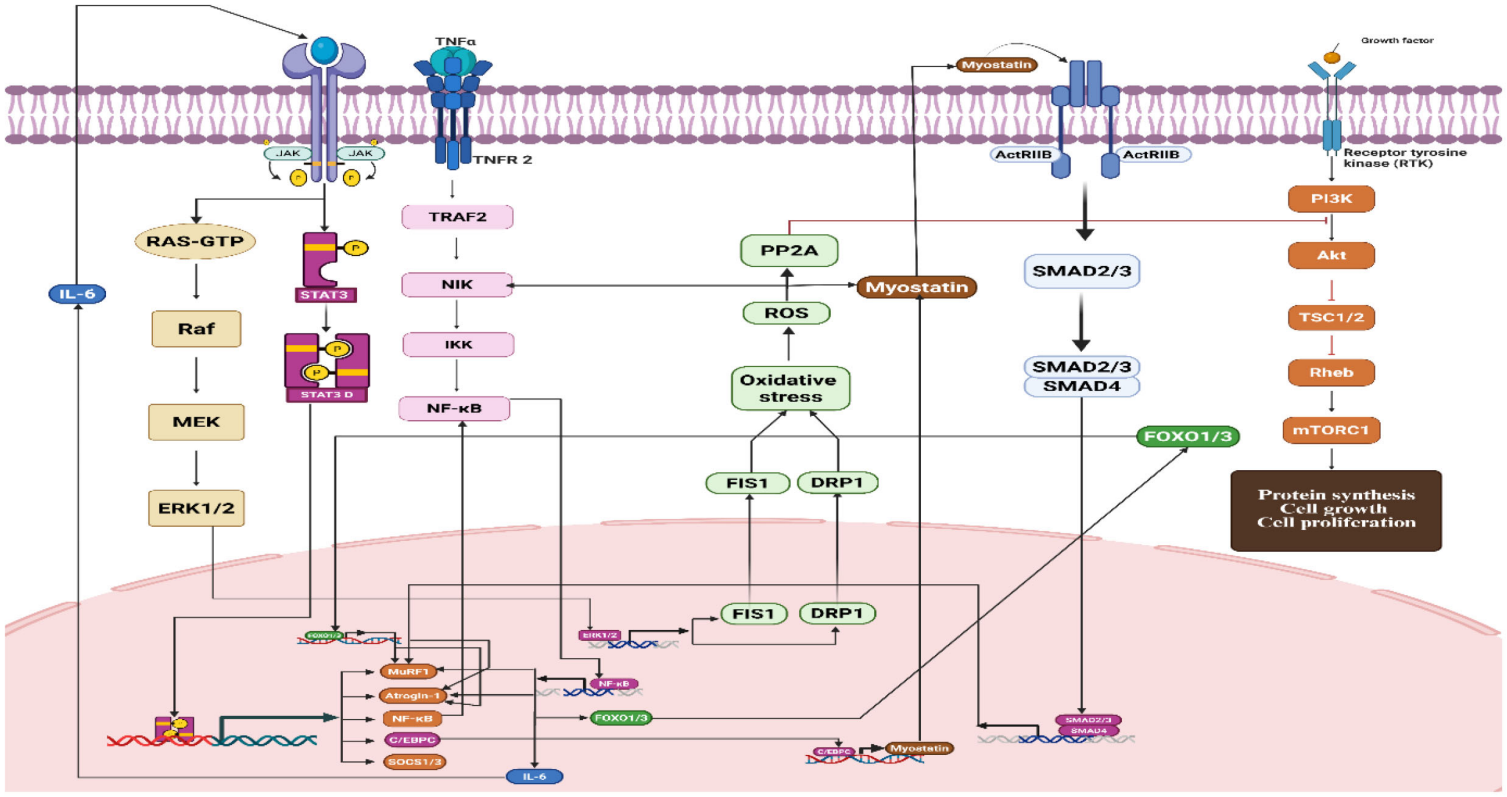
Reconstructed mathematical model of NSCLC induced sarcopenia.

To resolve the complexity of NSCLC-associated sarcopenia, we developed a mechanistic mathematical model integrating IL-6/JAK/STAT3, TNF-α/NF-κB, myostatin/SMAD2/3, oxidative stress–mitochondrial dynamics, and IGF-1/PI3K/AKT/mTORC1 signaling into a unified reaction network (**Figure 1**). The Flux analysis, crosstalk point mapping, and principal component analysis revealed that muscle wasting is associated with a compact regulatory network centered on IL-6 signaling, myostatin-SMAD2/3 activity, FOXO1/3 regulation, and the ubiquitin ligases MuRF1 and Atrogin-1. High-flux reactions, including IL-6 secretion and SMAD2/3-mediated induction of MuRF1 and Atrogin-1, exhibited prominent contributions to overall network behavior. Crosstalk point analysis further identified these reactions as key convergence points linking inflammatory and catabolic signaling pathways. Model reduction demonstrated that the principal characteristics of the network could be recapitulated using a limited set of highly influential components. Collectively, these findings support that inflammatory and catabolic signaling pathways converge on MuRF1 and Atrogin-1 to regulate muscle proteolysis in NSCLC-associated sarcopenia and provide a framework for future experimental investigation of these regulatory interactions.

## Materials and methods

This study employed a computational systems-biology framework to dissect the molecular mechanisms underlying non-small cell lung cancer (NSCLC) induced sarcopenia. The workflow integrated signaling network reconstruction, dynamic simulation, sensitivity analysis, principal component analysis (PCA), flux analysis, crosstalk point identification, and model reduction. The primary objective was to identify dominant regulatory components that govern inflammation-driven muscle proteolysis.

### Reconstruction of the sarcopenia signaling network

Through extensive literature curation, we reconstructed a comprehensive signaling network that captures how NSCLC driven inflammatory stress disrupts muscle homeostasis. This network integrates tumor-derived cytokine signaling with skeletal muscle proteostasis pathways and highlights several key mechanisms viz., inflammatory cascades such as IL-6/JAK/STAT3 and TNF-α/TNFR/NF-κB; negative regulators of muscle growth including Myostatin/ActRIIB/SMAD2-3-4; suppression of anabolic signaling via IGF-1/PI3K/Akt/mTORC1; transcriptional control by FOXO1/3, NF-κB, and STAT3; proteolytic machinery involving MuRF1 and Atrogin-1 within the ubiquitin–proteasome system; and oxidative stress with mitochondrial dynamics mediated by ROS, PP2A, FIS1, and DRP1. The network maps tumor–muscle cross-talk across extracellular, cytoplasmic, and nuclear compartments, with molecular interactions defined by experimentally validated activation, inhibition, binding, translocation, and transcriptional events reported in cancer cachexia, sarcopenia, and NSCLC studies. This integrative framework provides a systems-level view of how inflammatory–proteolytic signaling undermines muscle integrity in the context of NSCLC.

### Mathematical model formulation

We translated the reconstructed signaling network into a mechanistic mathematical model using ordinary differential equations (ODEs) within the SBML-based MATLAB SimBiology Toolbox (v7.11.1.866, The Mathworks Inc., Natick, MA, USA). This modeling environment enables dynamic modulation of kinetic rate constants and initial molecular abundances, providing a robust framework to simulate both homeostatic and pathological signaling states. Reaction kinetics was assigned according to biological context i.e., the law of mass action for association and dissociation events, Michaelis–Menten kinetics for phosphorylation, dephosphorylation, and enzymatic reactions, and Hill kinetics for transcriptional regulation of genes such as MuRF1 and Atrogin-1. Initial concentrations of cytokines, signaling proteins, and transcription factors were set within biologically realistic ranges reported in the literature which states an individual cell can release 10^3 to 10^6 molecules (45).


$$\text{d}\left[\text{Atrogin}-1\right]/\text{dt}={\text{f}}_{\text{STAT}3}+{\text{f}}_{\text{SMAD}2/3-4}+{\text{f}}_{\text{NF}-\kappa \text{B}}+{\text{f}}_{\text{FOXO}}-{\text{k}}_{\text{deg},\text{Atrogin}-1}\cdot \left[\text{Atrogin}-1\right]$$

$$\text{d}\left[\text{MuRF}1\right]/\text{dt}={\text{f}}_{\text{STAT}3}+{\text{f}}_{\text{SMAD}2/3-4}+{\text{f}}_{\text{NF}-\kappa \text{B}}+{\text{f}}_{\text{FOXO}}-{\text{k}}_{\text{deg},\text{MuRF}1}\cdot \left[\text{MuRF}1\right]$$

$$\text{d}\left[\text{FOXO}\right]/\text{dt}={\text{f}}_{\text{Akt inhibition}}-{\text{k}}_{\text{deg},\text{FOXO}}\cdot \left[\text{FOXO}\right]$$

$$\text{d}\left[\text{NF}-\kappa \text{B}\right]/\text{dt}={\text{f}}_{\text{pro}-\text{inflammatory signals}}-{\text{f}}_{\text{anti}-\text{inflammatory suppression}}-{\text{k}}_{\text{deg},\text{NF}-\kappa \text{B}}\cdot \left[\text{NF}-\kappa \text{B}\right]$$

Where each f term represents the Hill-type activation functions already defined in our model (e.g., STAT3-driven transcription) and the final term introduces first-order degradation proportional to the protein concentration.

Each activation term (f_STAT3_,f_SMAD2/3-4_,f_NF-κB,_f_FOXO_) represents Hill-type transcriptional induction functions already defined in the model, ensuring cooperative signaling dynamics are captured. To prevent unrealistic monotonic accumulation, a first-order degradation term proportional to protein concentration (−kdeg·[X]) was added for each species, reflecting basal proteasomal turnover and ubiquitin-mediated clearance. Feedback regulation was incorporated where supported by experimental evidence i.e., FOXO activity is negatively regulated by Akt-mediated phosphorylation and nuclear export, while NF-κB activity is counterbalanced by anti-inflammatory suppression through IκB and cytokines such as IL-10. This formulation ensures that protein levels rise and fall dynamically in response to upstream signals, rather than accumulating indefinitely, and provides a mechanistically credible framework for simulating muscle atrophy signaling.

Simulations were conducted for 100 time units using the ODE15s solver, which is well-suited for stiff systems and ensured stable, accurate solutions while keeping concentrations within biologically plausible ranges. This approach prevented numerical artifacts and allowed reliable tracking of dynamic changes. The behavior of molecules was further analyzed by plotting concentration profiles over time, which revealed pathway activity and cross-talk (46). Sensitivity analyses confirmed that the dominance of MuRF1 and Atrogin-1 persisted across wide parameter variations, demonstrating that this outcome reflects intrinsic network behavior rather than modeling bias. The complete mathematical model developed in MATLAB SimBiology is provided in **Supplementary Table 2**. The model comprises 46 biochemical reactions and 53 dynamic molecular species representing interconnected inflammatory, anabolic, and catabolic signaling pathways implicated in NSCLC-associated muscle wasting. All reaction equations, kinetic laws, parameter values, initial concentrations, and boundary conditions are provided in **Supplementary Table 1** (**S6**–**S8**).

### Sensitivity analysis

Sensitivity analysis was performed to evaluate the influence of individual parameters and molecular species on the dynamic behavior of the reconstructed sarcopenia signaling network. This analysis provides a quantitative measure of how small perturbations in reaction rates or initial concentrations propagate through the system and alter model outputs, thereby allowing identification of critical regulatory components within the network (47). Sensitivity coefficients were calculated in a time-dependent manner with respect to both kinetic parameters and species concentrations. The analysis was implemented using the MATLAB SimBiology environment, where sensitivities were computed simultaneously with the integration of the ordinary differential equations governing the model (45). The SUNDIALS-based deterministic solver was used to calculate local sensitivities by integrating auxiliary differential equations alongside the original system equations. Each parameter was perturbed within biologically relevant ranges while maintaining the structural integrity of the signaling network. Scaled sensitivity coefficients were obtained for all model components and reactions, generating a sensitivity matrix representing the contribution of each parameter to overall system behavior. This matrix served as the quantitative basis for subsequent multivariate analyses, including principal component analysis and model reduction. Parameters exhibiting minimal sensitivity were considered non-dominant and were retained only if necessary to preserve network stability, ensuring that later reduction steps did not disrupt system dynamics. All sensitivity calculations were performed under deterministic conditions using the same simulation settings applied during model construction to maintain consistency across computational analyses.

### Principal component analysis

Principal component analysis (PCA) is statistical method to reduce the dimensional complexity of the reconstructed sarcopenia signaling network and to identify dominant components governing system behavior. PCA was applied to the scaled sensitivity coefficient matrix generated during sensitivity analysis of the ODE-based model, where each element represented the influence of a specific molecular component or parameter on model output. The sensitivity matrix consisted of 53 model species and 53 sensitivity outputs (53 × 53 matrix). This approach enables extraction of key regulatory features by transforming high-dimensional sensitivity data into a smaller set of orthogonal variables that capture the majority of system variance (48). Sensitivity matrix was constructed using time-dependent local sensitivity coefficients obtained from deterministic simulations. The matrix was normalized and subjected to eigenvalue decomposition to generate principal components representing independent modes of variation within the network. PCA calculations were performed using MATLAB statistical functions, where the sensitivity matrix served as the input dataset. Components with high principal component scores were interpreted as major contributors to the overall network phenotype, whereas low-scoring elements were considered less influential for downstream model behavior (46).

The PCA framework enabled systematic identification of the principal signaling components governing network behavior without bias toward any individual pathway. By reducing the complexity of the sensitivity dataset into a smaller set of principal variables, PCA allowed clear separation of dominant inflammatory and proteolytic regulators from secondary signaling components with comparatively lower influence. This dimensionality reduction further provided a quantitative foundation for subsequent model reduction by identifying components whose exclusion would significantly affect overall network stability and alter system dynamics.

### Flux analysis

Flux analysis is used to quantify the contribution of individual reactions to the overall behavior of the reconstructed signaling network under steady-state conditions. This approach provides a reaction-level assessment of pathway activity by estimating the rate at which molecular species are converted through each biochemical step, thereby identifying reactions that exert dominant control over network output. In systems-level biological models, flux analysis is particularly useful for distinguishing highly active reactions that sustain phenotype-defining processes from those with minimal contribution to system dynamics (49). For the present model, flux values were calculated using COPASI (v4.35.258), which numerically solves the ordinary differential equations governing the reconstructed network and determines reaction rates based on species concentration and kinetic parameters. Each reaction flux was computed under the same deterministic conditions used during model simulation to ensure consistency across analyses. The resulting steady-state flux distribution enabled ranking of reactions according to their relative contribution within the network. This approach is appropriate because COPASI’s deterministic reaction-rate analysis provides internally consistent flux values that reflect the relative activity of pathways under the specified kinetic parameters, thereby enabling meaningful comparison and ranking even when absolute units are not directly interpretable biologically.

Reactions exhibiting higher flux values were interpreted as dominant transitions within the inflammatory-proteolytic signaling axis, indicating greater functional influence on muscle wasting progression. In contrast, reactions with low flux were considered less contributory to overall system behavior. This analysis provided an additional quantitative layer for identifying critical control points beyond sensitivity-based component ranking. Flux analysis was further integrated with sensitivity-derived information to support downstream model reduction. Reactions showing both low sensitivity and low flux were considered candidates for removal, whereas high-flux reactions were retained because of their significant contribution to network stability and phenotype preservation. This combined strategy enabled selective simplification of the signaling network while maintaining its essential biological behavior, consistent with previously established systems-biology modeling approaches.

### Model reduction

Model reduction was performed to simplify the reconstructed sarcopenia signaling network while preserving the essential biological characteristics of the system. Because large signaling networks often contain reactions and intermediate species that contribute minimally to the final phenotype, systematic reduction is necessary to improve computational efficiency, reduce parameter redundancy, and enhance interpretability without altering dominant network behavior.

The Model reduction was based on the integrated evaluation of sensitivity analysis, concentration, and flux analysis. Components and reactions that consistently exhibited low sensitivity values, low concentration, and low reaction flux were considered non-dominant and selected for potential elimination. In contrast, components with high influence across these analyses were retained because of their critical role in maintaining network dynamics (50). This combined approach ensured that only those reactions with minimal influence on overall system output were removed, while preserving biologically critical pathways. This stepwise reduction strategy allowed removal of redundant reactions while preserving the core inflammatory and proteolytic signaling structure of the model. The reduced model maintained stable system behavior and retained the major regulatory relationships observed in the full network, thereby providing a simplified but biologically representative framework for further analysis.

### Crosstalk point analysis

Crosstalk point analysis refers to identify shared regulatory components that connect multiple signaling pathways within the reconstructed sarcopenia network. In complex biological systems, signaling pathways do not function independently; instead, several molecular components serve as common interaction points through which different pathways converge and influence one another. Identifying such nodes is important because these shared components often exert disproportionate control over network behavior and may serve as potential intervention points for therapeutic modulation (51). In our study, crosstalk points were determined using a network topology-based approach in which the degree of each node was calculated across the complete signaling network and compared with its degree within individual pathway modules (52). A non-zero difference between these values was used to define a crosstalk component, indicating that the corresponding molecule participated in more than one signaling route and therefore contributed to pathway integration. Components with higher non-zero degrees values were considered stronger crosstalk points because of their broader influence across signaling modules. This analysis enabled identification of shared regulatory nodes that integrate multiple biological processes and provided an additional structural basis for prioritizing key components together with sensitivity, flux, and PCA results.

### Functional enrichment and network analysis

To investigate biological processes and signaling pathways linked to inflammation-driven proteostasis, we curated a list of key regulatory genes from our mathematical model and conducted functional enrichment analysis using g:Profiler (version e110_eg57_p18) (53). The analysis was performed across multiple annotation databases, including Gene Ontology Biological Processes (GO: BP), Kyoto Encyclopedia of Genes and Genomes (KEGG), and Reactome pathways. *Homo sapiens* were specified as the reference organism, and default statistical settings were applied with the g: SCS multiple testing correction method. Enriched terms were considered significant if they met the adjusted p-value (q-value) threshold of< 0.05. To enhance biological interpretability and relevance to muscle proteostasis, redundant and non-specific biological processes and pathways were manually curated and excluded. Pathways specifically associated with inflammatory signaling (e.g., IL-6/JAK–STAT), FOXO-mediated proteolysis, oxidative stress, and metabolic regulation were prioritized for downstream analysis. Filtered enrichment results were exported in Generic Enrichment Map format and visualized in Cytoscape using the EnrichmentMap plugin, where nodes represent pathways and edges indicate gene overlap. Networks were constructed using a q-value cutoff of ≤ 0.005–0.01 and a similarity coefficient ≥ 0.7, followed by layout optimization using Prefuse Force Directed or yFiles Organic algorithms. Functional modules were identified and annotated using AutoAnnotate and clusters were curated into biologically relevant groups (54).

### CytoHubba analysis and frequency of occurrence

CytoHubba is a Cytoscape plugin which was used to identify key nodes within the constructed signaling networks. Topological analyses were performed using twelve distinct algorithms, including degree, betweenness, closeness, eccentricity, radiality, stress, bottleneck, maximum neighborhood component (MNC), density of maximum neighborhood component (DMNC), edge percolated component (EPC), and maximal clique centrality (MCC). These measures collectively rank nodes based on their centrality and connectivity within the network. The frequency of occurrence of a species in the rank list of all the above-mentioned parameters were highlighted thereby identifying potential regulatory hubs that may play pivotal roles in modulating the inflammatory mediated proteostasis in NSCLC induced sarcopenia.

### Cell culture and transwell co-culture assay

To experimentally validate the computationally predicted inflammatory–proteolytic signaling events associated with NSCLC-induced sarcopenia, an *in vitro* co-culture system was established using murine skeletal muscle myoblasts C2C12 cells and human lung cancer cell lines. C2C12 cells were maintained under standard growth conditions in Dulbecco’s Modified Eagle Medium (DMEM) supplemented with 10% fetal bovine serum and 1% penicillin–streptomycin. Human lung cancer cell lines NCI-H1299, NCI-H1975, and A549 were cultured in parallel under their respective recommended growth conditions. Non-malignant bronchial epithelial BEAS-2B cells were included as the non-cancer control.

For co-culture experiments, 1 × 10^5^ C2C12 cells were seeded in six-well culture plates and allowed to adhere for 24 h. In parallel, 1 × 10^6^ lung epithelial or lung cancer cells were seeded into transwell inserts, maintaining a 1:10 ratio between muscle cells and epithelial/cancer cells. After stable attachment, transwell inserts containing the respective control or cancer cells were transferred into wells containing C2C12 cells, allowing indirect co-culture through soluble factor exchange without direct cell–cell contact. The co-culture system was maintained for 48 hours under standard incubator conditions (37 °C, 5% CO_2_). Experimental groups included C2C12 alone, C2C12 co-cultured with BEAS-2B, and C2C12 co-cultured separately with H1299, H1975, and A549 cells. This transwell-based design enabled evaluation of tumor-derived paracrine signaling effects on skeletal muscle cells while minimizing confounding effects from direct physical interaction.

### Enzyme-linked immunosorbent assay

The concentration of interleukin-6 (IL-6) in cell culture supernatants was quantified using a Human IL-6 ELISA Kit (KHC0061, Thermo Fisher Scientific, USA) according to the manufacturer’s instructions. Briefly, culture supernatants were collected from experimental groups and samples were added to the antibody-coated microplate wells. After that sample were mixed with Biotin-conjugated primary antibody and mixture was incubated for 2 h at room temperature on a shaker. The mix was discarded and the wells were washed four times with wash buffer. Following the washes, to each well 1:100 diluted streptavidin-HRP secondary antibody was added and incubated on shaker at RT for an hour. The wash buffer was then used three times to clean the wells. Following the addition of, Tetramethylbenzidine (TMB) substrate and 30 min of dark incubation, stop solution was added to the wells. A multi spectrophotometer was used to acquire the wells at 450 nm, and colorimetric measurements were recorded for every sample. By extrapolating on a standard curve graph, IL-6 levels were quantified.

### Confocal laser scanning microscopy

For immunofluorescence analysis, C2C12 cells from coculture experiment were seeded in 384 well coverslip-bottom plates and incubated for 24 h at 37 °C 5% CO2. Cells were fixed with 4% paraformaldehyde for 10 min at room temperature, followed by washing with 1× PBS. Permeabilization was carried out using 1× PBST (0.1% TritonX (9002-93–1 ThermoFischer Scientific) in 1X PBS) for 10 min at room temperature to allow intracellular antibody penetration. Non-specific binding sites were blocked with 3% bovine serum albumin prepared in PBST for 30 min at room temperature. Primary antibodies against MuRF1 and Atrogin-1 were diluted (1:1000) in blocking buffer and incubated with the cells for 2 h at room temperature. After primary incubation, samples were washed three times with PBST to remove excess antibody. The cells were incubated with anti-rabbit IgG (H +L) F (ab’)2 fragment (AlexaFluor 488 Conjugate) (4412S) Cell Signaling Technology) and phalloidin AlexaFluor 568 ((A12380) ThermoFisher Scientific) (1:500) for 1 h. Following secondary antibody incubation, cells were washed three times with PBST and counterstained with DAPI for 10 min to visualize nuclei. After final washing, samples were rinsed with distilled water and imaged using an Olympus FV3000 laser confocal microscope at 60× magnification. Identical acquisition settings were maintained for all experimental groups to ensure consistency in fluorescence comparison. Image analysis and fluorescence quantification were performed using Fiji (version 1.54f).

### Bulk transcriptomics

Four-week-old female immunodeficient mice (NOD-SCID) were maintained in a specific pathogen-free (SPF) animal facility under standard housing conditions. To establish the orthotopic lung cancer model, human non-small cell lung carcinoma cells H1299 were cultured under standard conditions and prepared for implantation. Mice were anaesthetised via intraperitoneal injection with a mixture of ketamine (100 mg/kg) and xylazine (4 mg/kg). Total 1 × 10^6^ H1299 cells suspended in 100 µL sterile phosphate-buffered saline (PBS) were administered via intrapulmonary injection into the right lung lobe. Following tumor cell implantation, mice were closely monitored until full recovery and subsequently observed daily for general health and signs of tumour progression. After 20 days post-implantation, mice were euthanized using Carbon dioxide (CO2) gas asphyxiation method as per approval from Institutional Animal Ethics Committee (IAEC) of NCCS. A gradual-fill method is mandated & the CO2 flow rate is set to displace 30% to 70% of the chamber’s volume per minute as per AVMA (American Veterinary Medical Association) Guidelines for the Euthanasia of Animals (2020 edition). After that 70% ethanol was used to disinfect the body. The mice skin was cut using scissors to reveal the peritoneum, and then forceps were used to reveal the lungs. Using forceps and scissors, the lung tissues were excised aseptically, rinsed in ice-cold PBS to remove residual blood, and immediately preserved in RNAlater stabilization solution to ensure RNA integrity. Samples were stored under appropriate conditions until further processing.

Preserved lung tissue samples were subjected to bulk RNA sequencing to investigate transcriptional alterations associated with tumor development. High-quality RNA samples were processed for next-generation sequencing, followed by downstream bioinformatic analysis to quantify gene expression and identify molecular pathways associated with inflammation and muscle proteostasis.

The raw fastq reads were preprocessed using Fastp v.0.23.4 (parameters: --trim_poly_g --cut_front --cut_tail --average_qual 30) (55). The rRNA reads were filtered from the trimmed fastq files using RiboDetector v0.3.1 (parameters: -e rrna) (56). The rRNA filtered reads were aligned to the STAR indexed *Homo sapiens* reference genome (GCF_000001405.40; source: NCBI) using STAR aligner v 2.7.9a (57). The alignment file (BAM) from individual samples were quantified using featureCounts v. 2.0.1 based on the GTF file of the reference, to obtain gene counts (58). Differential expression estimation was done based on the gene counts using EdgeR exectTest (59) (parameters: threshold of statistical significance --alpha 0.05; p-value adjustment method: BH). The ‘regularized log’ transformation in EdgeR exactTest was used for principal component and clustering analysis. The annotated EdgeR exactTest result file was filtered based on Adjusted p-value (FDR)<= 0.05 and LogFoldChange ∓ 1. The volcano plots were generated using EnhancedVolcano (60) and the MA plots were plotted using the ggmaplot function of ggpubr R package (61). Significantly differentially expressed transcripts were subjected to Gene Ontology (GO) and KEGG enrichment analysis using ShinyGO (62), with *Mus musculus* genome used as reference model, a significance threshold of 0.05, and False Discovery Rate (FDR) correction.

### Statistical analysis

All quantitative data are presented as mean ± SD. For confocal microscopy experiments, three independent biological experiments were performed for each condition (n = 3 biological replicates). In each experiment, fluorescence intensity was measured from five cells within a representative microscopic field using ImageJ software. Cell-level measurements were averaged to generate a single value for each biological replicate prior to statistical analysis. Statistical comparisons among groups were performed using one-way ANOVA followed by Dunnett’s multiple-comparison test, using C2C12 cells as the control group. Homogeneity of variance was assessed using the Brown–Forsythe test. P values< 0.05 were considered statistically significant.

## Results

### Reconstructed mathematical model and dynamic simulation reveal a dominant inflammatory–proteolytic network in NSCLC-induced sarcopenia

**Figure 2:**
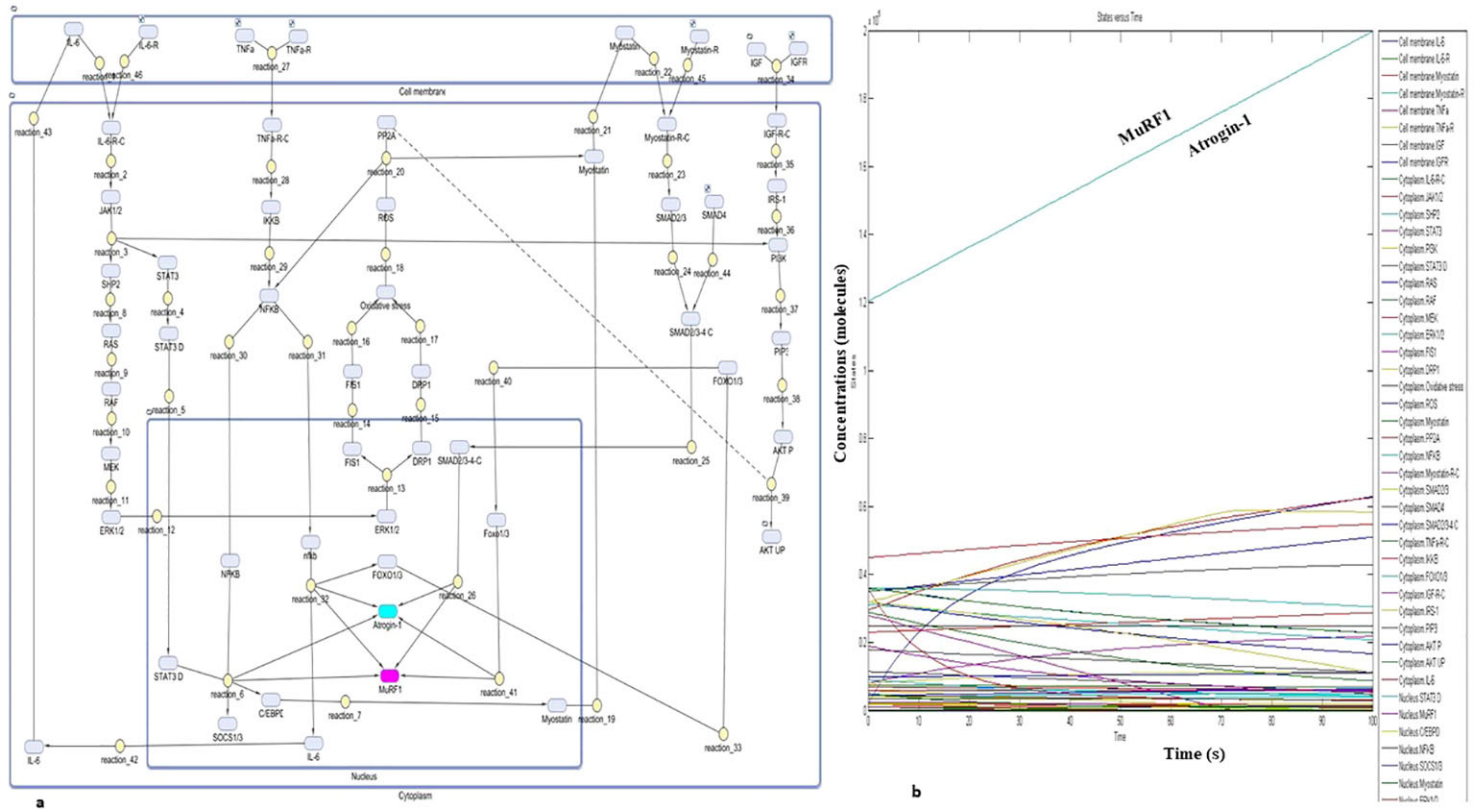
**(A)** Reconstructed signaling network describing the regulatory interactions governing NSCLC-induced sarcopenia, highlighting the IL-6/STAT3, TNF-α/NF-κB, Myostatin/SMAD, and IGF-1/PI3K/Akt pathways converging on MuRF1 and Atrogin-1. **(B)** Dynamic simulation of the mathematical model showing the temporal concentration profiles of the principal signaling molecules. The simulation demonstrates progressive activation of MuRF1 and Atrogin-1 downstream of inflammatory signaling.

To mechanistically understand the molecular basis of NSCLC-induced sarcopenia, a signaling network was reconstructed by integrating the major inflammatory, oxidative, anabolic, and proteolytic pathways known to regulate skeletal muscle homeostasis under tumor-associated stress. The model incorporated extracellular cytokine and ligand inputs including IL-6, TNF-α, myostatin, and IGF, together with their respective receptors positioned at the cell membrane, followed by intracellular signaling cascades involving JAK1/2–STAT3, TNFα–IKKβ–NF-κB, ROS-mediated oxidative stress signaling, myostatin–SMAD2/3/4, and IGF–PI3K–Akt pathways. These signaling modules converged within cytoplasmic and nuclear compartments to regulate transcriptional mediators such as FOXO1/3, C/EBP, and NF-κB, ultimately controlling the expression of the ubiquitin ligases MuRF1 and Atrogin-1, which represent effector molecules of muscle protein degradation. The reconstructed model was organized into three major compartments- cell membrane, cytoplasm, and nucleus, to preserve the spatial sequence of receptor activation, intracellular signal propagation, and transcriptional output. Within this framework, inflammatory signals initiated through IL-6 receptor activated STAT3-dependent signaling, while TNF-α signaling promoted NF-κB activation through IKKβ. In parallel, myostatin signaling through SMAD2/3/4 and suppression of anabolic signaling through IGF–PI3K–Akt created a coordinated catabolic environment. Oxidative stress components including ROS, PP2A, FIS1, and DRP1 were incorporated to capture mitochondrial stress contributions that further amplify proteolytic signaling (**Figure 2A**). The model was submitted to the Biomodel database with identifier MODEL2606150001. (sarco1 | BioModels).

Dynamic simulation of the reconstructed network generated time-dependent concentration profiles for all signaling components under steady-state progression. Among all simulated species, MuRF1 and Atrogin-1 exhibited the strongest progressive accumulation over time, emerging as the dominant component of the network (**Figure 2B**). Their sustained increase indicates that multiple upstream inflammatory and catabolic pathways converge robustly on ubiquitin–proteasome activation, suggesting that muscle protein degradation represents the most stable phenotypic outcome of prolonged tumor-derived signaling. The simulation further showed that although several upstream signaling intermediates displayed moderate temporal changes, only a restricted subset of downstream proteolytic regulators reached dominant concentrations, indicating hierarchical signal compression within the network. This behavior suggests that NSCLC-associated sarcopenia is not driven by uniform activation of all pathways, but rather by selective amplification of key terminal catabolic effectors. Collectively, the reconstructed model establishes that inflammatory cytokine signaling oxidative stress, and anabolic suppression form an integrated regulatory architecture in which MuRF1 and Atrogin-1 function as major endpoint molecules determinants of muscle wasting.

### Principal component analysis

To identify the dominant molecular components governing network behavior, principal component analysis (PCA) was performed using the sensitivity coefficient matrix derived from the reconstructed signaling model. This dimensionality reduction approach enabled separation of highly influential components from less contributory signaling elements by ranking each molecular species according to its principal component score. The first principal component (PC1) accounted for 86.28% of the total variance, indicating that a limited number of signaling components dominated system dynamics. PC2 and PC3 explained an additional 4.26% and 1.67% of the variance, respectively, resulting in a cumulative variance of 92.21% for the first three principal components.

Loading analysis was subsequently performed to identify the components contributing most strongly associated with system variation. Components exhibiting the highest absolute loading values are MuRF1 (Nu), IL-6 (Nu), STAT3 (C), DRP1 (Nu), SMAD4 (C), SMAD2/3-4-C (Nu), NF-κb (C), Myostatin (C), IL-6-R-C (C) and Foxo1/3 (Nu), with corresponding loading values of 0.4477, 0.4463, 0.4463, 0.4094, 0.4094, 0.1764, 0.0726, 0.0717, 0.0649, and 0.0519, respectively. As PC1 captured the majority of the total variance, these components were prioritized for biological interpretation and were considered key determinants of the regulatory architecture underlying NSCLC-associated sarcopenia (**Supplementary Table 1**).

**Figure 3:**
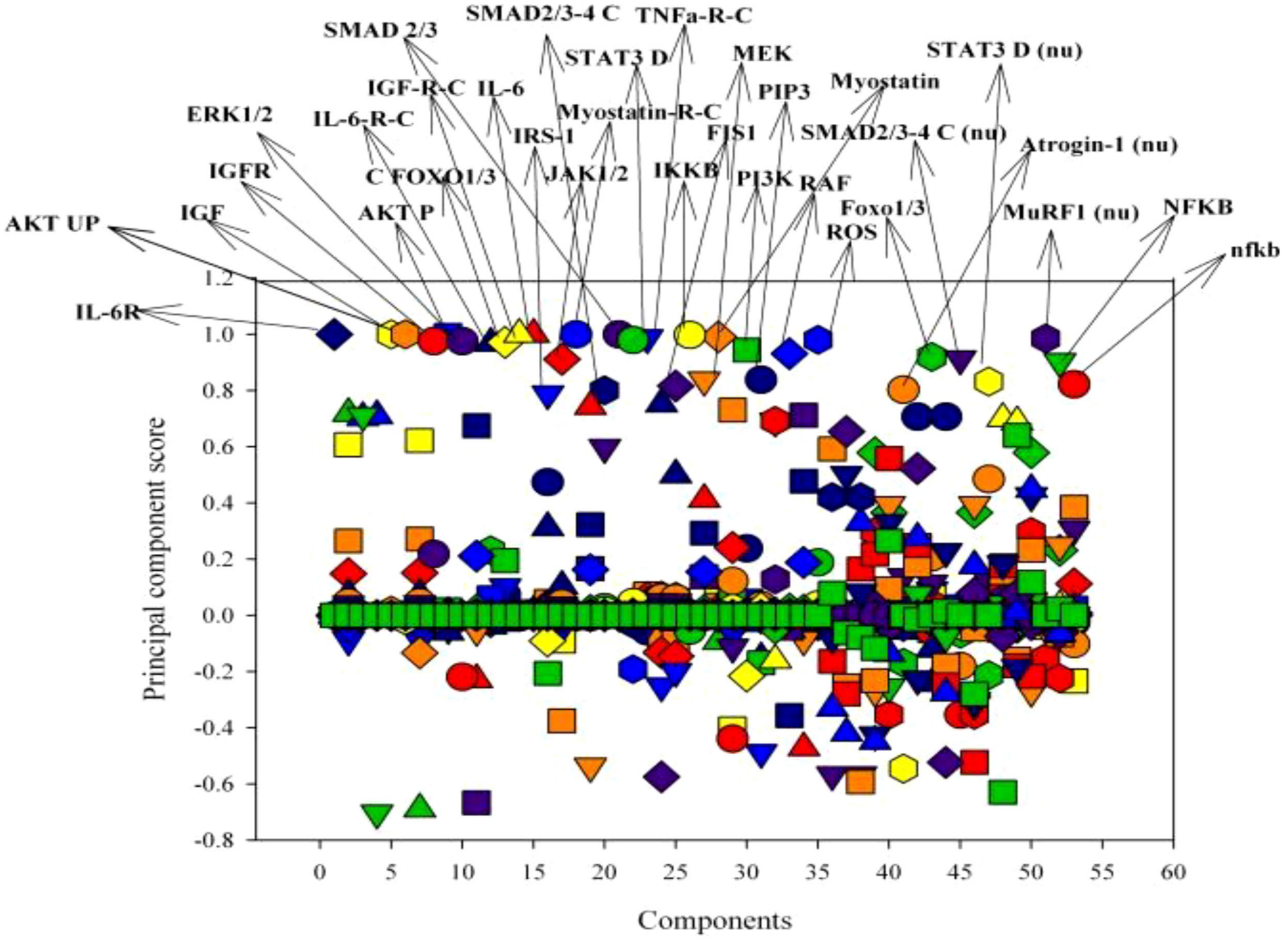
Principal component analysis of the sarcopenia network. Principal component scores derived from the sensitivity matrix highlighting dominant regulators of system behavior. High-scoring components include IL-6/STAT3, myostatin/SMAD, and FOXO1/3-driven proteolytic pathways, with MuRF1 and Atrogin-1 emerging as key determinants of network output.

The PCA distribution revealed that only a restricted subset of signaling molecules contributed strongly to the overall variance of the system, indicating that the sarcopenic phenotype is controlled by a compact regulatory core rather than by uniform participation of all modelled components. Components with highest principal component scores were predominantly associated with inflammatory signaling, anabolic suppression, and proteolytic regulation. Among the highest-ranking components, IL-6 signaling intermediates, IL-6R-C, and STAT3, emerged as major contributors, indicating that inflammatory cytokine signaling forms one of the principal axes controlling network behavior (**Figure 3**). In parallel, myostatin signaling components including myostatin, SMAD4, SMAD2/3, and SMAD2/3–4 complexes also exhibited strong principal scores, and contributes substantially to phenotype stabilization. Notably, downstream proteolytic regulators including Foxo1/3, and MuRF1 also occupied high-scoring positions, indicating that terminal ubiquitin–proteasome components are not merely downstream outputs but integral determinants of overall system variance.

### Flux analysis

Flux distribution analysis revealed that sarcopenic progression is driven by a small set of dominant biochemical processes rather than uniform activity across the network. The highest flux was observed for cytoplasmic-to-extracellular IL-6 transport (2979.7 mol/sec), underscoring sustained inflammatory signaling as a primary driver. Myostatin signaling also showed strong activity, with high flux linked to its secretion (577.2 mol/sec) and subsequent SMAD2/3 activation (397.16 mol/sec), reflecting robust propagation of catabolic signals. Downstream, the SMAD2/3–4 complex–mediated induction of MuRF1 and Atrogin-1 (381.98 mol/sec) emerged as one of the most active reactions, highlighting activation of the ubiquitin–proteasome pathway as a major functional output (**Table 1**). Additional contributions came from FOXO1/3 shuttling (~359 mol/sec) and NF-κB activation (280.97 mol/sec), pointing to strong transcriptional regulation of muscle atrophy genes. Overall, the flux analysis demonstrates that IL-6–driven inflammation, myostatin–SMAD signaling, and FOXO/NF-κB–mediated transcription converge to sustain high proteolytic activity, positioning MuRF1 and Atrogin-1 as central effectors of NSCLC-induced sarcopenia.

**Table 1:** High flux reactions from the reconstructed signaling model.

Species	Flux (mol/sec)
IL-6 (Cytoplasm) -> IL-6 (CM)	2979.7
Myostatin (C) -> Myostatin (CM)	577.2
Myostatin-R-C-> SMAD2/3	397.163
FIS1 (Nucleus) -> FIS1 (C)	390.478
SMAD2/3-4-C-> MuRF1 + Atrogin-1	381.988
FOXO1/3 (Nucleus) -> FOX01/3 (C)	359.98
FOXO1/3 (Cytoplasm) -> Foxo1/3	359.28
IL-6 (Nucleus) -> IL-6 (C)	359.2
NFKB (Cytoplasm) -> nfkb	280.971
Foxo1/3 -> MuRF1 + Atrogin-1	239.995

### Model reduction

**Figure 4:**
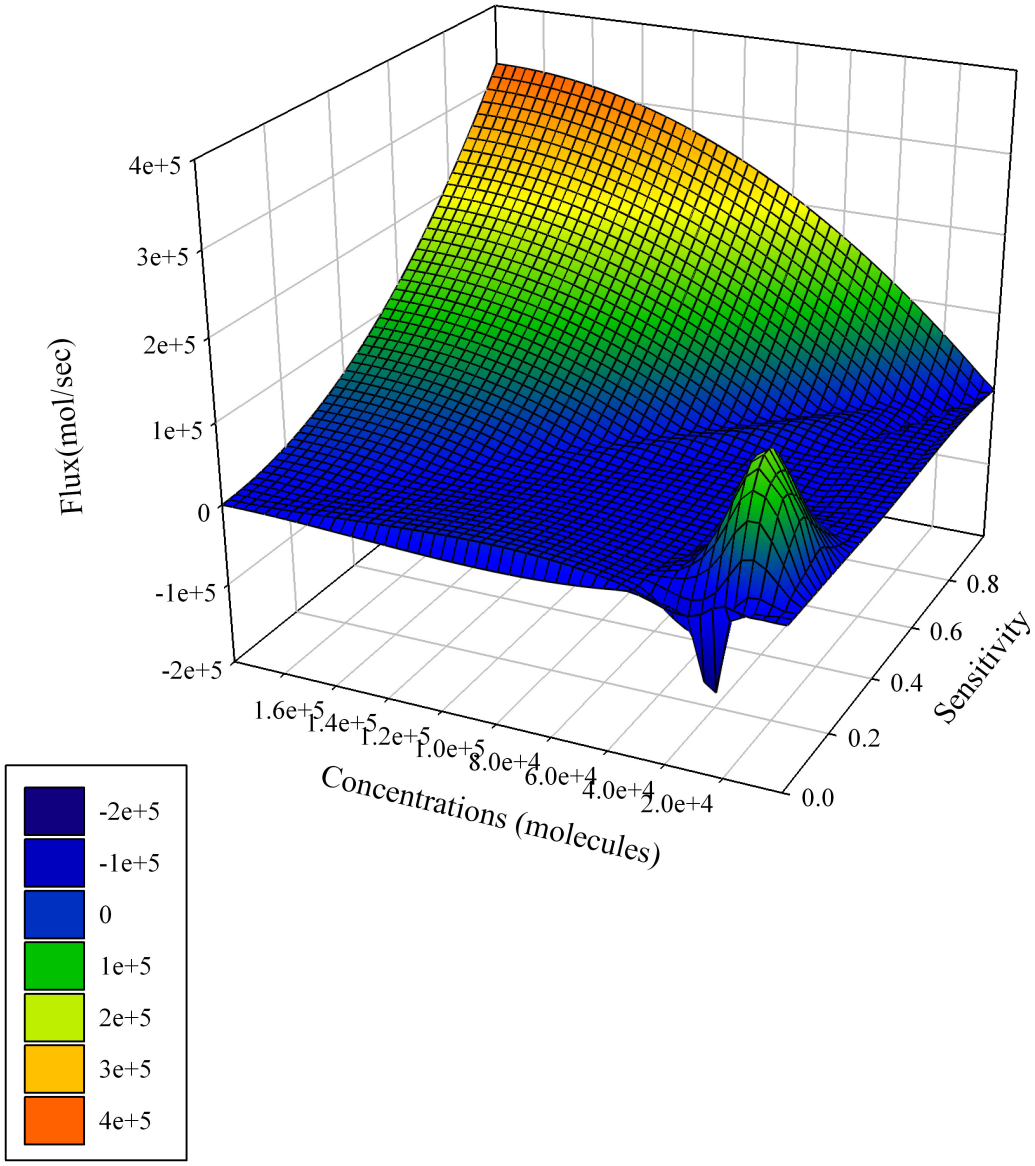
Quasi-potential landscape of the sarcopenia network. Three-dimensional surface plot showing the relationship between component concentration, sensitivity, and reaction flux. High-flux regions form a dome-like peak, indicating dominant reactions governing system behavior, while low-flux regions represent minimally contributing components used for model reduction.

To reduce model complexity while preserving key component, model reduction was performed by integrating results from sensitivity, concentrations and flux analysis. This combined approach enabled identification of reactions that significantly contribute to network output while excluding less influential components. The resulting quasi-potential landscape represented as a three-dimensional mesh, exhibited a dome-shaped structure in which components with high flux, high sensitivity and high concentration were localized at the peak. In contrast, components with low contribution were distributed toward the lower regions of the landscape (**Figure 4**). This distribution indicates that only a subset of reactions actively governs system dynamics, whereas a large proportion of reactions contributes minimally. Based on this analysis, reactions with low sensitivity and low flux were systematically removed, leading to a substantial reduction in model dimensionality. The retained reactions correspond to key phenotype-determining processes representing the minimal regulatory framework required to reproduce the sarcopenic behavior of the system.

### Crosstalk point analysis identifies key regulatory hubs

**Figure 5:**
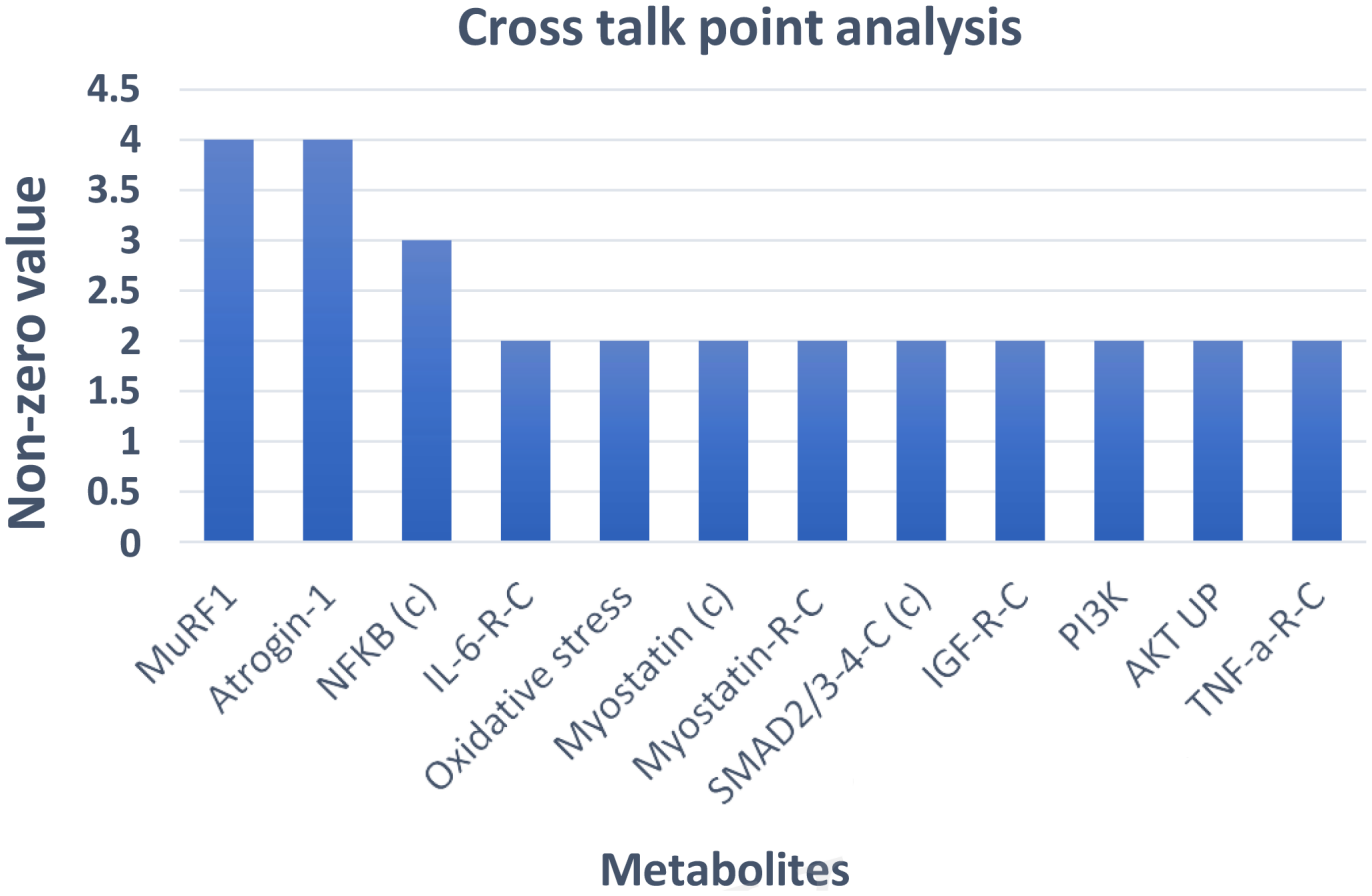
Crosstalk analysis identifies key regulatory hubs in the sarcopenia network. Bar plot showing non-zero connectivity values of network components. MuRF1 and Atrogin-1 exhibit the highest crosstalk, followed by NF-κB and intermediate signaling nodes, highlighting their roles as central integrators of inflammatory and proteolytic pathways.

Crosstalk point analysis revealed that only a small subset of components functions as major regulatory hubs within the reconstructed signaling network. Among these, MuRF1 and Atrogin-1 exhibited the highest crosstalk values, underscoring their central role as convergence points for multiple upstream pathways. This finding highlights that these ubiquitin ligases are not simply downstream effectors but act as key integrators of inflammatory, oxidative, and transcriptional signals driving muscle atrophy. NF-κB also showed relatively high crosstalk, reflecting its importance in linking inflammatory signaling with transcriptional regulation of atrophy-related genes. Moderate crosstalk values were observed for the IL-6 receptor complex, oxidative stress mediators, and myostatin signaling elements, suggesting their role as intermediate connectors between cytokine signaling and proteolytic activation. Interestingly, anabolic signaling components such as IGF receptor complexes, PI3K, and AKT contributed to network crosstalk as well, reflecting their interplay with catabolic pathways and emphasizing the balance between anabolic suppression and proteolytic activation in sarcopenic progression. TNFα receptor-associated signaling further reinforced pathway integration through its connection with NF-κB-mediated transcription (**Figure 5**). Together, these results highlight a layered regulatory architecture in which a few key hubs orchestrate the complex tumor–muscle cross-talk characteristic of NSCLC-associated sarcopenia.

### Functional enrichment and pathway network analysis reveal convergence on proteostasis disruption

**Figure 6:**
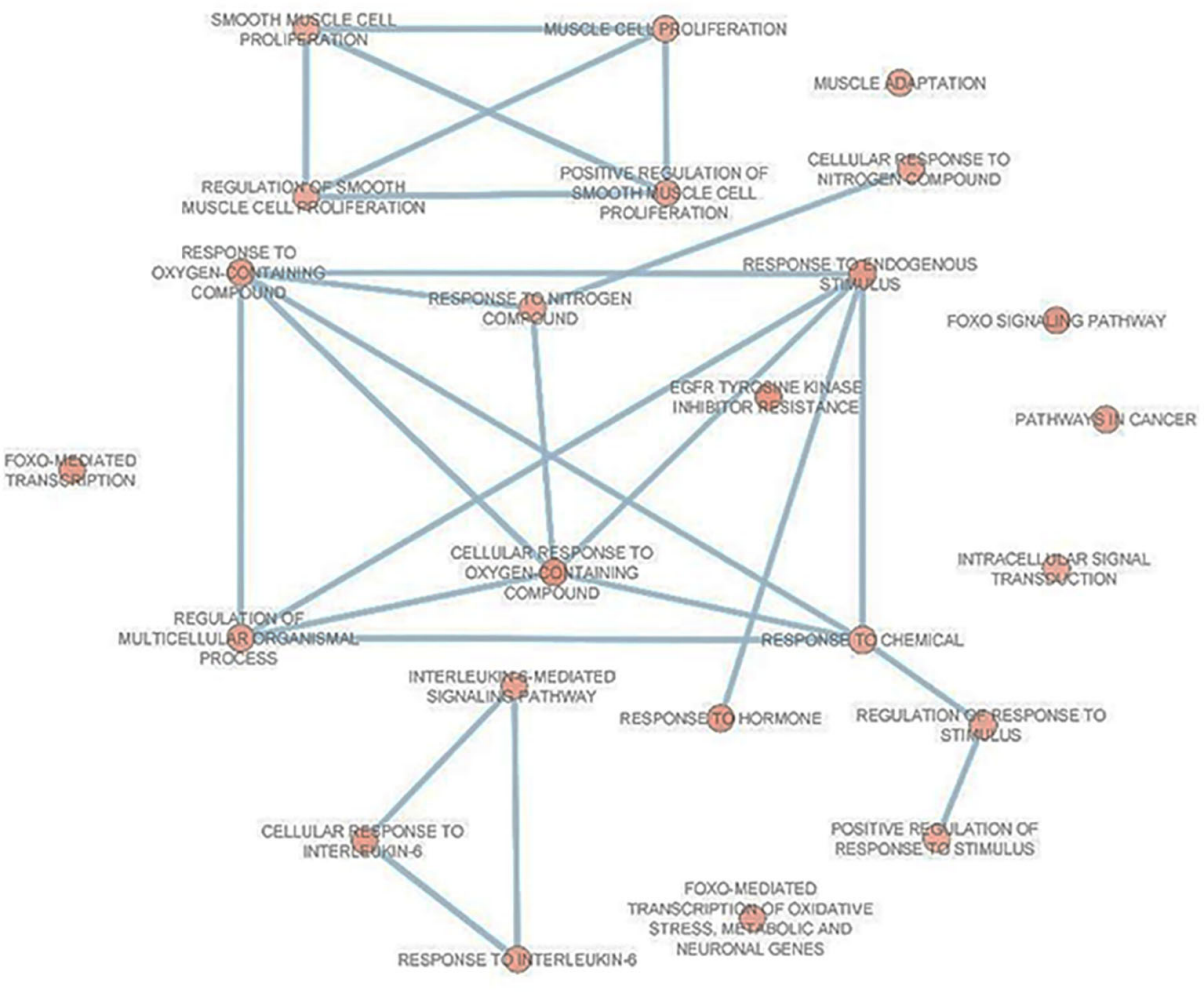
Enrichment map of biological processes regulating inflammation-driven muscle proteostasis. Nodes represent enriched GO biological processes and edges denote gene overlap. The network highlights coordinated pathways involving cytokine signaling, FOXO-mediated transcription, oxidative stress and muscle adaptation, collectively underpinning inflammatory regulation of muscle proteostasis.

To further validate the systems-level relevance of the reconstructed network, functional enrichment analysis was performed using g:Profiler, followed by pathway-level interpretation. The analysis revealed significant enrichment of biological processes associated with cellular response to cytokines, oxidative stress, and metabolic stimuli, with particularly strong enrichment for interleukin-6–mediated signaling (Padj< 10^-^¹¹) (**Figure 6**). Gene ontology analysis demonstrated enrichment in processes related to cellular response to oxygen-containing compounds, hormonal signaling, and muscle adaptation, indicating coordinated regulation of stress and metabolic pathways. Notably, IL-6 response and cytokine-mediated signaling emerged as dominant biological processes, supporting the central role of inflammatory signaling in sarcopenic progression.

Pathway enrichment analysis (KEGG) identified key signaling pathways, including FoxO signaling, PI3K–Akt signaling, TNF signaling and JAK–STAT signaling, all of which are directly associated with muscle protein turnover and inflammatory regulation. In parallel, Reactome analysis highlighted FOXO-mediated transcription, interleukin signaling, and SMAD/TGF-β signaling pathways, further confirming the integration of inflammatory and catabolic signaling axes. Importantly, network-level interpretation of enriched pathways revealed that these signaling modules are not independent but form a highly interconnected regulatory system. Inflammatory pathways (IL-6/STAT3, TNF/NF-κB) converge with catabolic regulators (FOXO, SMAD2/3) and anabolic signaling suppression (PI3K–Akt), collectively promoting activation of ubiquitin–proteasome components such as MuRF1 and Atrogin-1 (**Supplementary Table 1** (**S5**)).

### Network-based identification of central hub regulators in inflammation-mediated muscle proteostasis

**Figure 7:**
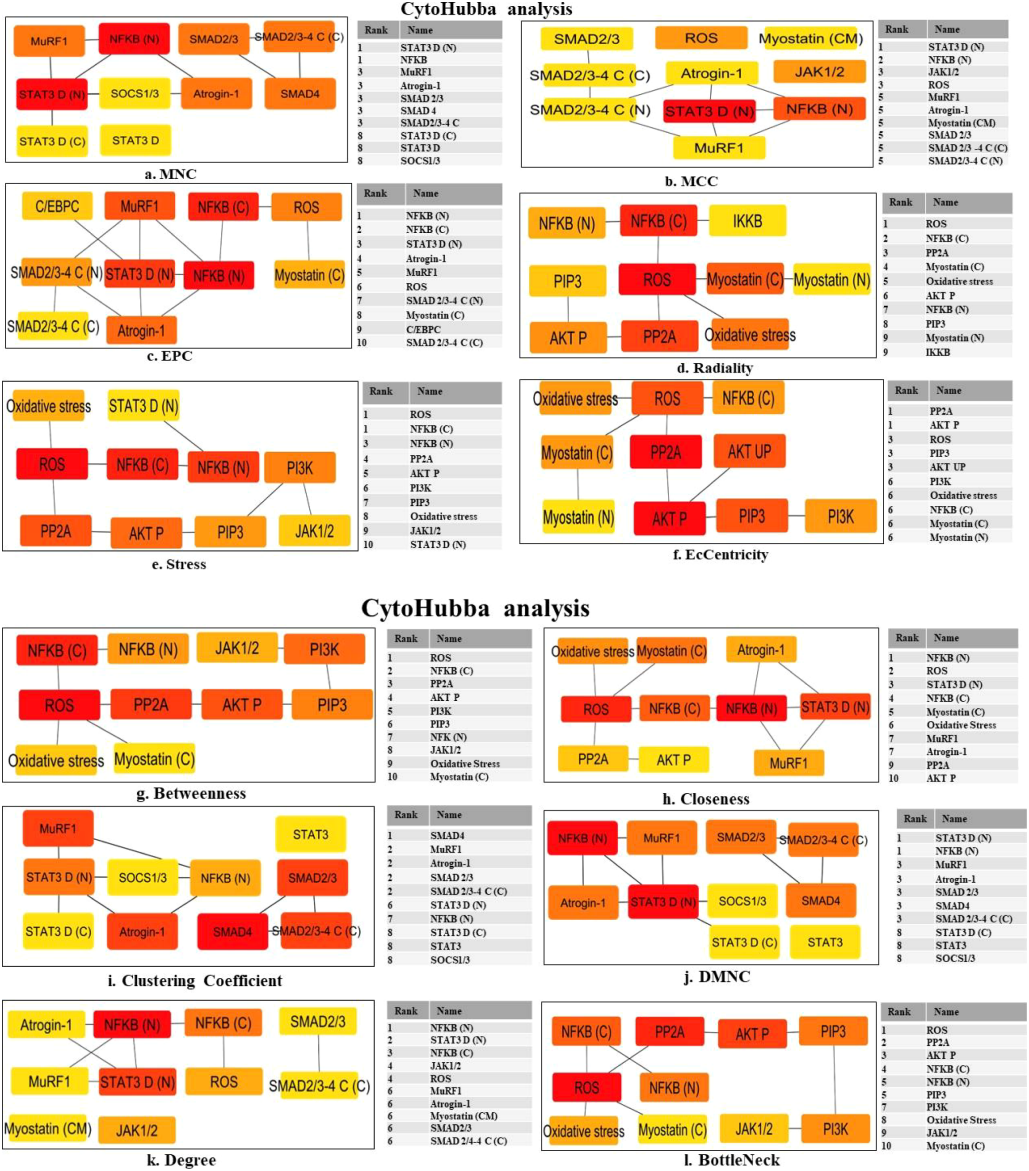
CytoHubba analysis of the network **(A)** MNC, **(B)** MCC, **(C)** EPC, **(D)** Radiality, **(E)** Stress, **(F)** Eccentricity, **(G)** Betweenness, **(H)** Closeness, **(I)** Clustering Co-efficient, **(J)** DMNC, **(K)** Degree, **(L)** BottleNeck.

**Figure 8:**
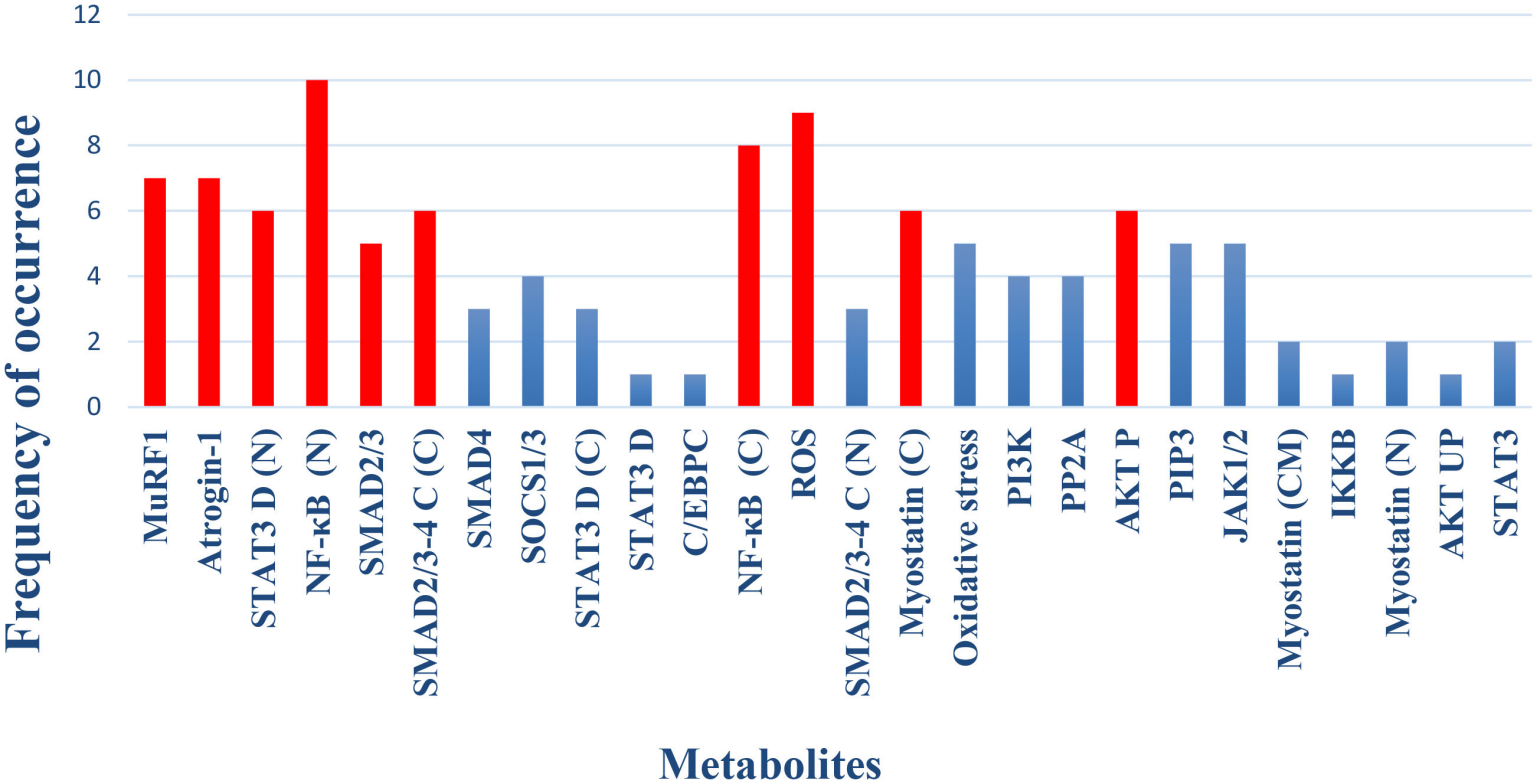
Frequency of occurrence plot of network components evaluated based on CytoHubba parameters. The components highlighted in red are top 10 components with frequency of occurrence in 5 or more than 5 parameters.

The network’s top-ranking nodes were identified using a variety of topological analysis techniques using CytoHubba. Total 12 parameters were used to identify key nodes based on MCC, MNC, DMNC, EPC, radiality, degree, proximity, betweenness, eccentricity, bottleneck, clustering coefficient, and stress centrality (**Figure 7**). The analysis revealed that NF-κB, ROS, MuRF1 and Atrogin-1 exhibited the highest occurrence scores, confirming their dominant role as central executors of muscle protein degradation (**Figure 8**). The NF-κB and STAT3 highlighting their critical contribution to inflammation-mediated transcriptional control of atrophy-related genes. Additionally, components of the myostatin axis, including SMAD2/3 and myostatin-associated complexes, demonstrated consistent representation, indicating their involvement in promoting catabolic signaling and suppressing muscle growth. Oxidative stress–associated nodes (ROS) also displayed elevated frequency, supporting their role in amplifying proteolytic pathways. Conversely, anabolic signaling components such as PI3K, AKT, and PIP3 were moderately represented, suggesting a functional imbalance favoring catabolic dominance under inflammatory conditions. The presence of intermediates such as JAK1/2, IKKβ, and PP2A further emphasizes the integration of cytokine signaling with intracellular regulatory cascades. Collectively, these findings define a hierarchical network in which inflammatory signaling (NF-κB/STAT3), stress responses (ROS), and myostatin–SMAD pathways converge to activate ubiquitin–proteasome machinery via MuRF1 and Atrogin-1, ultimately driving muscle proteolysis.

### IL-6 secretion is elevated in lung cancer cell co-culture conditions

To investigate the inflammatory response associated with lung cancer-induced muscle wasting, IL-6 levels were quantified from supernatants collected from C2C12 (Control) and C2C12:Beas2B, C2C12:H1299 C2C12:H1975 and C2C12:A549 co-culture conditions. Significant differences in IL-6 secretion were observed all the lung cancer cell line groups. Basal IL-6 production was negligible in C2C12 cells (3.9 pg/mL) and BEAS-2B cells (4.7 pg/mL). In contrast, lung cancer cell lines exhibited markedly elevated IL-6 concentrations, with H1299, H1975, and A549 cells producing 287.7, 266.1, and 284.9 pg/mL IL-6, respectively. These findings indicate that lung cancer cells secrete substantially higher levels of IL-6 compared with non-tumorigenic controls, supporting a potential role for IL-6-mediated inflammatory signaling in cancer-associated muscle wasting.

### Confocal microscopy

**Figure 9:**
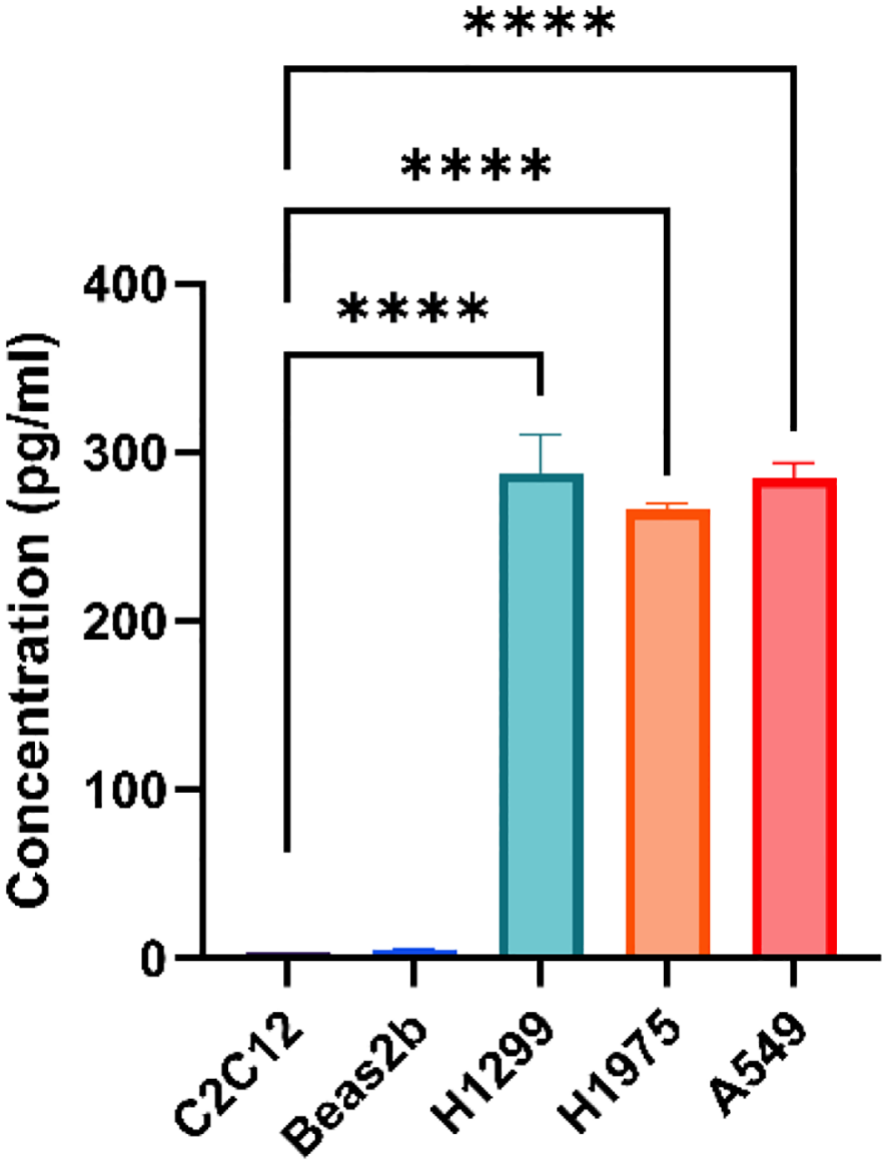
Elevated IL-6 secretion by lung cancer cells. IL-6 concentrations in culture supernatants were quantified using a human IL-6 ELISA kit (Thermo Fisher Scientific, KHC0061). Supernatants were collected from C2C12, BEAS-2B bronchial epithelial cells, and lung cancer cell lines (H1299, H1975, and A549). Data are presented as mean ± SD from two independent experiments (n = 2). Statistical analysis was performed using one-way ANOVA followed by Dunnett’s multiple-comparison test with C2C12 as the control group. H1299, H1975, and A549 cells exhibited significantly higher IL-6 secretion compared with C2C12 cells (****P< 0.0001), whereas BEAS-2B cells showed no significant difference (ns).

**Figure 10:**
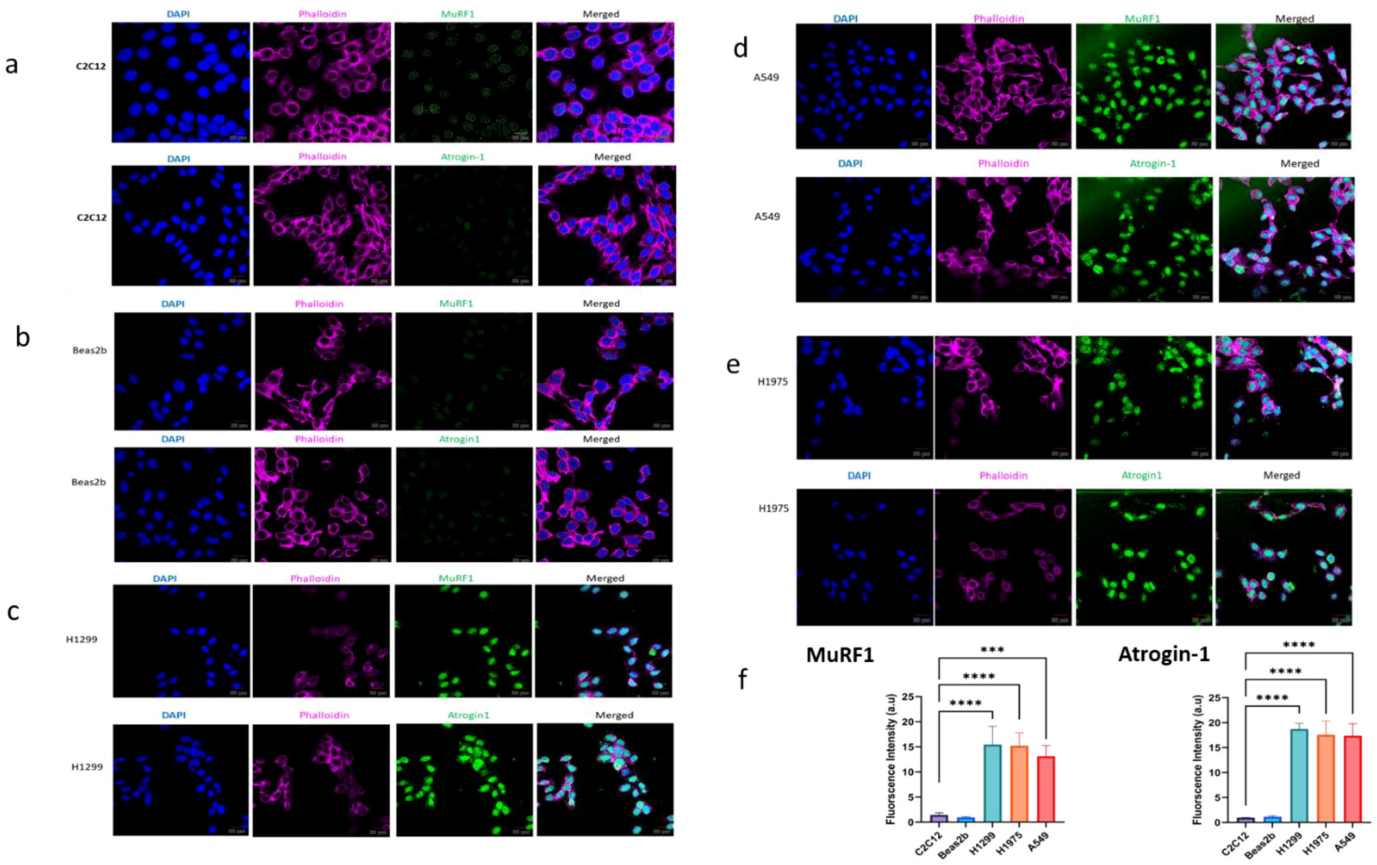
Confocal immunofluorescence analysis of muscle atrophy markers in C2C12 myotubes following co-culture with lung epithelial and NSCLC cells. Representative confocal images showing nuclei (DAPI, blue), F-actin (phalloidin, magenta), and MuRF1 or Atrogin-1 (green) in **(A)** C2C12 control, **(B)** Beas-2B co-culture, **(C)** H1299 co-culture, **(D)** A549 co-culture, and **(E)** H1975 co-culture conditions. Merged images demonstrate enhanced expression of MuRF1 and Atrogin-1 in NSCLC co-culture groups compared with controls. **(F)** Quantification of fluorescence intensity revealed significant upregulation of both MuRF1 and Atrogin-1 under cancer co-culture conditions. Data are presented as mean ± SEM from three independent biological experiments (n = 3 per group). Fluorescence intensity was quantified by averaging measurements from five cells per experiment. Statistical significance was assessed using one-way ANOVA followed by Dunnett's multiple-comparison test against the C2C12 control group (***P < 0.001, ****P < 0.0001).

To experimentally validate the computational predictions of proteolytic pathway activation, we employed immunofluorescence-based confocal microscopy to examine MuRF1 and Atrogin-1 expression and localization in C2C12 cells under co-culture conditions. Compared with control C2C12 cells, co-culture with lung cancer cell lines consistently produced a marked increase in fluorescence intensity for both proteins, indicating enhanced activation of muscle atrophy-associated pathways (**Figures 9**, **10**). In contrast, C2C12 cells co-cultured with non-malignant epithelial cells displayed expression levels comparable to baseline, underscoring the tumor-specific effect. Subcellular analysis revealed pronounced accumulation of MuRF1 and Atrogin-1 in cytoplasmic and perinuclear regions, consistent with active engagement of the ubiquitin–proteasome system. Together, these findings demonstrate that soluble factors derived from lung cancer cells promote proteolytic signaling in skeletal muscle corroborating the mechanistic insights predicted by the computational model.

### Bulk transcriptomics

**Figure 11:**
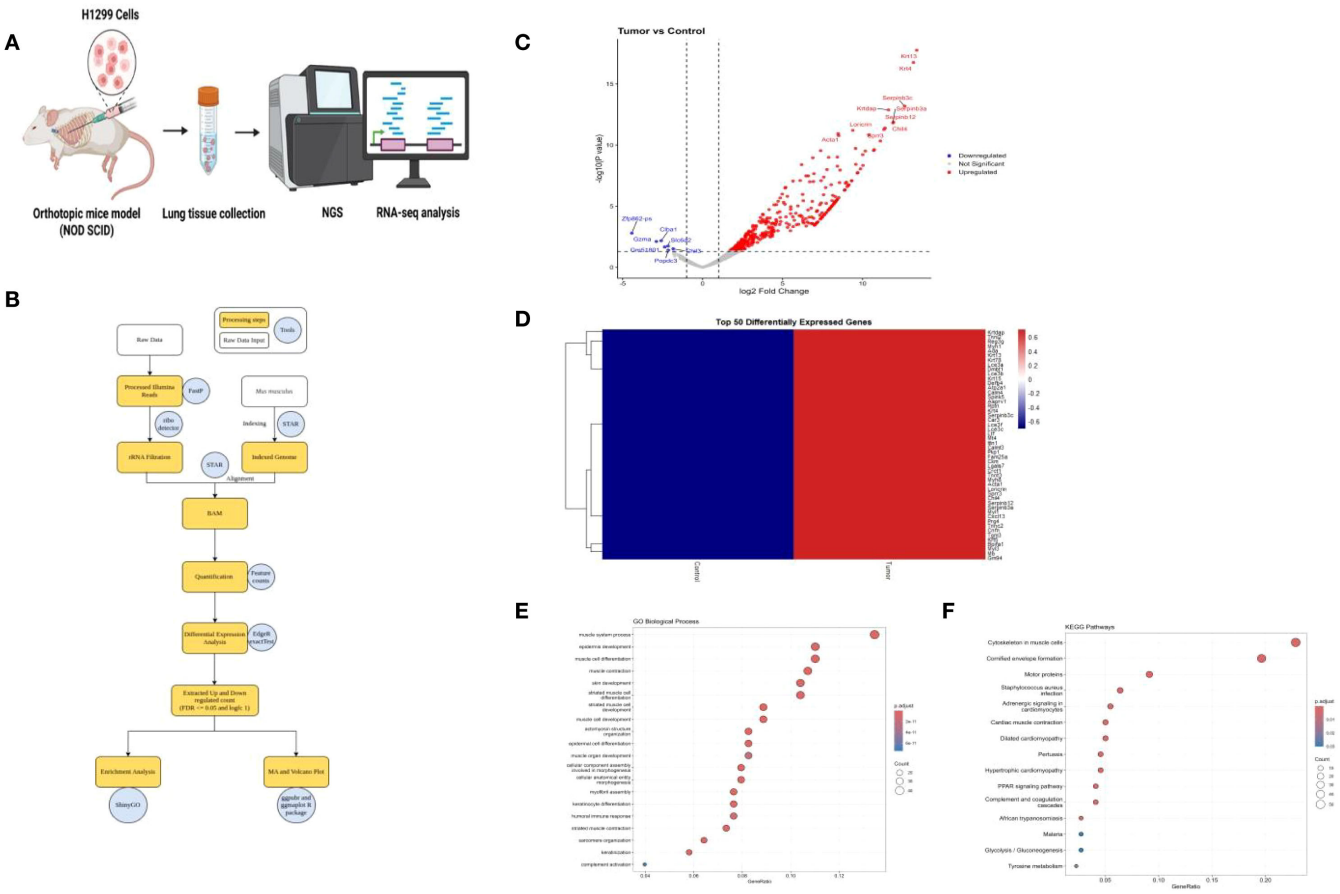
**(A)** Orthotopic lung cancer model generated by intrapulmonary injection of H1299 cells in NOD-SCID mice, followed by lung tissue collection and RNA-seq analysis. **(B)** Overview of the experimental data analysis workflow- Pipeline adopted for analysis of bulk transcriptomics data. **(C)** (Volcano plot of expressed genes of study groups. The *y* axis illustrates −log 10 *p* values, and the *x* axis corresponds to a logfold change of gene expression between Study groups. The red points represent statistically significant genes (FDR< 0.05). The genes represented in black are non-significant (NS) and green (logFC) points denote the ones that were not statistically significant and were not considered for downstream analyses. **(D)** Heatmap of the top 50 differentially expressed genes distinguishing tumor and control samples. **(E)** Functional enrichment showing the top most significant (FDR<0.05) categories of biological processes (GO terms) based on up and down regulated genes for study (The top GO Biological Processes are plotted in order of Fold change. The size of the dots represents the number of genes in the significant DE gene list associated with the GO term and the colour of the dots represents the Fold Enrichment). **(F)** Functional enrichment showing the top most significant (FDR<0.05) categories of KEGG terms based on up and down regulated genes for study (The top KEGG are plotted in order of Fold change. The size of the dots represents the number of genes in the significant DE gene list associated with the KEGG term and the color of the dots represents the Fold Enrichment).

Bulk transcriptomic analysis of tumor versus normal lung tissue in mice revealed enrichment of cytoskeletal and muscle-development pathways, offering a mechanistic window into how NSCLC may contribute to muscle wasting (**Figure 11**). In lung cancer, systemic inflammation and tumor-derived factors are known to activate transcriptional programs that upregulate muscle-specific ubiquitin ligases such as MuRF1 and Atrogin-1, accelerating proteasomal degradation of myofibrillar proteins. Concurrently, elevated myostatin signaling suppresses anabolic pathways like Akt/mTOR, impairing muscle regeneration and growth. The enrichment of cytoskeletal remodeling genes may therefore reflect stress responses consistent with atrophy, in line with the clinical phenotype of cachexia in NSCLC patients. These molecular changes highlight why sarcopenia is a frequent and debilitating comorbidity in NSCLC, worsening prognosis, reducing treatment tolerance, and underscoring the need for therapeutic strategies that target proteolysis, myostatin signaling, and inflammatory drivers of muscle loss. However, because the RNA-seq analysis was performed on lung tissue from the orthotopic H1299 model rather than skeletal muscle, these findings should be regarded as hypothesis-generating rather than definitive evidence of sarcopenia. Direct assessment of skeletal muscle and functional atrophy measures will be required to substantiate this link.

## Discussion

Our systems-level analysis highlights a compact yet hierarchical signaling network that orchestrates muscle proteolysis in NSCLC-associated sarcopenia. Topological ranking of nodes using CytoHubba revealed NF-κB, ROS, MuRF1, and Atrogin-1 as dominant executors of protein degradation, underscoring their centrality in the catabolic program. NF-κB and STAT3 emerged as critical transcriptional regulators of atrophy-related genes, while the myostatin–SMAD axis consistently reinforced catabolic signaling and suppression of muscle growth. Elevated representation of oxidative stress associated nodes (ROS) further supports their role in amplifying proteolytic cascades, whereas anabolic mediators such as PI3K, AKT and PIP3 were only moderately represented, reflecting a functional imbalance favoring catabolism under inflammatory conditions.

The convergence of inflammatory (IL-6/STAT3, TNFα/NF-κB), catabolic (myostatin/SMAD) and proteolytic (FOXO1/3, MuRF1, Atrogin-1) pathways defines a tightly integrated regulatory core. This architecture suggests that sarcopenia is not driven by isolated pathway activation but by coordinated signaling that sustains muscle protein breakdown. Experimental validation using confocal microscopy confirmed increased expression and altered localization of MuRF1 and Atrogin-1 in cancer co-culture conditions, providing biological support for the computational predictions. The bulk transcriptomics analysis showing enrichment of cytoskeletal and muscle development pathways in NSCLC lungs dovetails directly with the experimental findings of MuRF1 and Atrogin-1 dysregulation. At the transcriptome level, the tumor-bearing lung tissue reveals a shift in gene expression toward pathways that govern muscle structure and remodeling, consistent with a systemic catabolic state. This computational signal aligns with the biological validation.

By reconstructing a comprehensive signaling network and translating it into a mechanistic ODE-based model, this study advances our understanding of NSCLC-induced sarcopenia beyond descriptive observations. The framework not only identifies critical nodes and flux transitions that quantitatively define muscle wasting behavior but also offers a versatile platform for drug discovery. Collectively, these findings establish a systems-level perspective on the molecular underpinnings of cancer-associated muscle loss and open avenues for therapeutic interventions aimed at restoring proteostasis.

## Limitations and future perspectives

The proposed systems biology framework provides mechanistic insights into lung cancer-associated sarcopenia; however, several limitations should be acknowledged. The model has not yet been validated in independent patient cohorts, and the lack of skeletal-muscle-specific transcriptomic data limits direct validation of the predicted mechanisms. In addition, model calibration relied on limited perturbation datasets, while some kinetic parameters were derived from literature sources or estimated during model development, introducing uncertainty into quantitative predictions. The framework also does not fully capture species-specific differences or the heterogeneity of tumor, immune, and skeletal muscle cell populations.

Future integration of patient-derived multi-omics data and patient-specific parameters may improve predictive accuracy and clinical relevance. Extending the framework to other cancer types and cachexia-related conditions could further uncover shared and disease-specific regulatory mechanisms, supporting precision medicine approaches.

## Conclusions

In conclusion, integrative analysis reveals that NSCLC-induced sarcopenia is orchestrated by a compact yet hierarchical signaling network in which inflammatory, catabolic and proteolytic pathways converge to sustain muscle protein breakdown. Bulk transcriptomics highlight cytoskeletal and muscle remodeling signatures, while mechanistic modeling and experimental validation pinpoint NF-κB, STAT3, ROS, MuRF1 and Atrogin-1 as dominant executors of proteolysis. Our study has unveiled that systems-level perspective moves beyond descriptive associations and provides a platform for therapeutic discovery aimed at improving patient survivability in future.

## Data Availability

The original contributions presented in the study are included in the article/**Supplementary Material**. Further inquiries can be directed to the corresponding author.
