# Supplementary material for: IL-6 signaling orchestrates proteolytic hubs MuRF1 and Atrogin-1 in NSCLC induced sarcopenia
Source: Front Immunol. 2026 Aug 3;17:1885023. doi: 10.3389/fimmu.2026.1885023 (PMC13478115; doi:10.3389/fimmu.2026.1885023)
Supplement: Supplementary file 1 [file Table2.docx]

<?xml version="1.0" encoding="UTF-8"?>

<sbml xmlns="http://www.sbml.org/sbml/level2" xmlns:html="http://www.w3.org/1999/xhtml" level="2" version="1">

<annotation>

Created by The MathWorks, Inc. SimBiology tool, Version 3.3

</annotation>

<model id="mw1c89e1f8_40da_475c_a64b_ebb9a34a6295" name="sarco1">

<listOfUnitDefinitions>

<unitDefinition id="MWBUILTINUNIT_molecule" name="molecule">

<listOfUnits>

<unit kind="mole" multiplier="1.66053872801495e-024" offset="0"/>

</listOfUnits>

</unitDefinition>

<unitDefinition id="MWDERIVEDUNIT_1__second" name="1/second">

<listOfUnits>

<unit kind="second" exponent="-1" multiplier="1" offset="0"/>

<unit kind="dimensionless" multiplier="1" offset="0"/>

</listOfUnits>

</unitDefinition>

<unitDefinition id="MWDERIVEDUNIT_molecule__second" name="molecule/second">

<listOfUnits>

<unit kind="mole" multiplier="1" offset="0"/>

<unit kind="second" exponent="-1" multiplier="1" offset="0"/>

<unit kind="dimensionless" multiplier="1.66053872801495e-024" offset="0"/>

</listOfUnits>

</unitDefinition>

<unitDefinition id="MWDERIVEDUNIT_1__molecule_second" name="1/(molecule*second)">

<listOfUnits>

<unit kind="mole" exponent="-1" multiplier="1" offset="0"/>

<unit kind="second" exponent="-1" multiplier="1" offset="0"/>

<unit kind="dimensionless" multiplier="6.02214199e+023" offset="0"/>

</listOfUnits>

</unitDefinition>

</listOfUnitDefinitions>

<listOfCompartments>

<compartment id="mw11138c27_1850_44ac_a5b4_d9750c52ad7c" name="Cell membrane" size="1"/>

<compartment id="mwc0549e25_5531_4b14_9196_afc5b3c40236" name="Cytoplasm" size="1"/>

<compartment id="mw4fe466a8_8f95_4dde_8a8b_18d4b7ad73b0" name="Nucleus" size="1" outside="mwc0549e25_5531_4b14_9196_afc5b3c40236"/>

</listOfCompartments>

<listOfSpecies>

<species id="mwea1c0c15_2b67_46fb_929b_7c1267ad9963" name="IL-6" compartment="mw11138c27_1850_44ac_a5b4_d9750c52ad7c" initialAmount="1600" substanceUnits="MWBUILTINUNIT_molecule" hasOnlySubstanceUnits="true"/>

<species id="mwcbdd87f9_b3ed_4d0a_9e54_0fdbf6fcde76" name="IL-6-R" compartment="mw11138c27_1850_44ac_a5b4_d9750c52ad7c" initialAmount="1000" substanceUnits="MWBUILTINUNIT_molecule" hasOnlySubstanceUnits="true" boundaryCondition="true"/>

<species id="mw8b46f320_13f4_457c_bd6f_e2cea1cd0e03" name="Myostatin" compartment="mw11138c27_1850_44ac_a5b4_d9750c52ad7c" initialAmount="29511" substanceUnits="MWBUILTINUNIT_molecule" hasOnlySubstanceUnits="true"/>

<species id="mwca4fe501_c33f_471c_9ec9_b4ef623dd41a" name="Myostatin-R" compartment="mw11138c27_1850_44ac_a5b4_d9750c52ad7c" initialAmount="25000" substanceUnits="MWBUILTINUNIT_molecule" hasOnlySubstanceUnits="true" boundaryCondition="true"/>

<species id="mwc0a9a42d_48d4_468f_bab9_574edacbe15a" name="TNFa" compartment="mw11138c27_1850_44ac_a5b4_d9750c52ad7c" initialAmount="2000" substanceUnits="MWBUILTINUNIT_molecule" hasOnlySubstanceUnits="true" boundaryCondition="true"/>

<species id="mwfbd3d650_d9b8_4fe3_9643_0a891d49c063" name="TNFa-R" compartment="mw11138c27_1850_44ac_a5b4_d9750c52ad7c" initialAmount="2000" substanceUnits="MWBUILTINUNIT_molecule" hasOnlySubstanceUnits="true" boundaryCondition="true"/>

<species id="mwed30a3ca_aba6_4030_9bb8_0ad3fff03228" name="IGF" compartment="mw11138c27_1850_44ac_a5b4_d9750c52ad7c" initialAmount="200" substanceUnits="MWBUILTINUNIT_molecule" hasOnlySubstanceUnits="true" constant="true"/>

<species id="mwee2376de_9c09_486e_af20_4eeb71df78d1" name="IGFR" compartment="mw11138c27_1850_44ac_a5b4_d9750c52ad7c" initialAmount="1000" substanceUnits="MWBUILTINUNIT_molecule" hasOnlySubstanceUnits="true" boundaryCondition="true"/>

<species id="mw37536c65_bf58_4c51_a2ee_d0100642163f" name="IL-6-R-C" compartment="mwc0549e25_5531_4b14_9196_afc5b3c40236" initialAmount="980" substanceUnits="MWBUILTINUNIT_molecule" hasOnlySubstanceUnits="true"/>

<species id="mw154f7171_cdd5_408d_b100_5d2d4faa8c9b" name="JAK1/2" compartment="mwc0549e25_5531_4b14_9196_afc5b3c40236" initialAmount="1778" substanceUnits="MWBUILTINUNIT_molecule" hasOnlySubstanceUnits="true"/>

<species id="mw486ef628_30a7_422a_b579_d3aa2c3f2fd4" name="SHP2" compartment="mwc0549e25_5531_4b14_9196_afc5b3c40236" initialAmount="3400" substanceUnits="MWBUILTINUNIT_molecule" hasOnlySubstanceUnits="true"/>

<species id="mwb67146fa_d038_4406_920d_414761f272e0" name="STAT3" compartment="mwc0549e25_5531_4b14_9196_afc5b3c40236" initialAmount="2375" substanceUnits="MWBUILTINUNIT_molecule" hasOnlySubstanceUnits="true"/>

<species id="mw16a20824_e3b6_4f7b_aa76_6a31fe8cbb0c" name="PI3K" compartment="mwc0549e25_5531_4b14_9196_afc5b3c40236" initialAmount="5841" substanceUnits="MWBUILTINUNIT_molecule" hasOnlySubstanceUnits="true"/>

<species id="mwdf1f48ca_5474_4a18_a9ac_accc2ea8d515" name="STAT3 D" compartment="mwc0549e25_5531_4b14_9196_afc5b3c40236" initialAmount="3368" substanceUnits="MWBUILTINUNIT_molecule" hasOnlySubstanceUnits="true"/>

<species id="mwbfbf065a_5322_4a5e_8f93_6cd8ffb41ea7" name="RAS" compartment="mwc0549e25_5531_4b14_9196_afc5b3c40236" initialAmount="7000" substanceUnits="MWBUILTINUNIT_molecule" hasOnlySubstanceUnits="true"/>

<species id="mw89b78fe1_5fdd_4290_8d1e_0cea4ac30f64" name="RAF" compartment="mwc0549e25_5531_4b14_9196_afc5b3c40236" initialAmount="7500" substanceUnits="MWBUILTINUNIT_molecule" hasOnlySubstanceUnits="true"/>

<species id="mwf9e54da4_564e_460f_aee4_28eb334e5fe9" name="MEK" compartment="mwc0549e25_5531_4b14_9196_afc5b3c40236" initialAmount="5900" substanceUnits="MWBUILTINUNIT_molecule" hasOnlySubstanceUnits="true"/>

<species id="mw88274cec_267a_4a84_9cc3_de3d0eaac1cb" name="ERK1/2" compartment="mwc0549e25_5531_4b14_9196_afc5b3c40236" initialAmount="6955" substanceUnits="MWBUILTINUNIT_molecule" hasOnlySubstanceUnits="true"/>

<species id="mw759859f4_f3b3_48e7_bf82_740506b598b0" name="FIS1" compartment="mwc0549e25_5531_4b14_9196_afc5b3c40236" initialAmount="7998" substanceUnits="MWBUILTINUNIT_molecule" hasOnlySubstanceUnits="true"/>

<species id="mweb909918_2d84_489e_a6e4_a8b51c6b2040" name="DRP1" compartment="mwc0549e25_5531_4b14_9196_afc5b3c40236" initialAmount="7998" substanceUnits="MWBUILTINUNIT_molecule" hasOnlySubstanceUnits="true"/>

<species id="mw34aeb325_8500_4f88_8835_0d633dbea428" name="Oxidative stress" compartment="mwc0549e25_5531_4b14_9196_afc5b3c40236" initialAmount="17896" substanceUnits="MWBUILTINUNIT_molecule" hasOnlySubstanceUnits="true"/>

<species id="mwfd54e35c_b0e8_4374_b666_981e29d936c6" name="ROS" compartment="mwc0549e25_5531_4b14_9196_afc5b3c40236" initialAmount="35000" substanceUnits="MWBUILTINUNIT_molecule" hasOnlySubstanceUnits="true"/>

<species id="mw1e8b346c_6576_495e_9457_539c8f2a3023" name="Myostatin" compartment="mwc0549e25_5531_4b14_9196_afc5b3c40236" initialAmount="28860" substanceUnits="MWBUILTINUNIT_molecule" hasOnlySubstanceUnits="true"/>

<species id="mwf3f1e954_4fa7_4d92_b277_2062ac96263a" name="PP2A" compartment="mwc0549e25_5531_4b14_9196_afc5b3c40236" initialAmount="23000" substanceUnits="MWBUILTINUNIT_molecule" hasOnlySubstanceUnits="true"/>

<species id="mw26bfd5c7_b3a6_4615_8b41_7ac6a7c78c1b" name="NFKB" compartment="mwc0549e25_5531_4b14_9196_afc5b3c40236" initialAmount="31219" substanceUnits="MWBUILTINUNIT_molecule" hasOnlySubstanceUnits="true"/>

<species id="mw0fb4af26_aaa9_46cf_820f_a01414045446" name="Myostatin-R-C" compartment="mwc0549e25_5531_4b14_9196_afc5b3c40236" initialAmount="28000" substanceUnits="MWBUILTINUNIT_molecule" hasOnlySubstanceUnits="true"/>

<species id="mwab852ab4_c2c5_4647_a2ac_b14e2642d48b" name="SMAD2/3" compartment="mwc0549e25_5531_4b14_9196_afc5b3c40236" initialAmount="31865" substanceUnits="MWBUILTINUNIT_molecule" hasOnlySubstanceUnits="true"/>

<species id="mw1c2ae8fe_bef4_408b_a731_b47363bf4c63" name="SMAD4" compartment="mwc0549e25_5531_4b14_9196_afc5b3c40236" initialAmount="25000" substanceUnits="MWBUILTINUNIT_molecule" hasOnlySubstanceUnits="true" boundaryCondition="true"/>

<species id="mw3d1df92d_2bbf_45fa_8039_0f7a0a668b47" name="SMAD2/3-4 C" compartment="mwc0549e25_5531_4b14_9196_afc5b3c40236" initialAmount="31864" substanceUnits="MWBUILTINUNIT_molecule" hasOnlySubstanceUnits="true"/>

<species id="mw2552df9f_943e_4f52_a55d_cf0e98b255c3" name="TNFa-R-C" compartment="mwc0549e25_5531_4b14_9196_afc5b3c40236" initialAmount="980" substanceUnits="MWBUILTINUNIT_molecule" hasOnlySubstanceUnits="true"/>

<species id="mwa847064b_ceba_41e5_b9c8_0a8f98dc781a" name="IKKB" compartment="mwc0549e25_5531_4b14_9196_afc5b3c40236" initialAmount="1772" substanceUnits="MWBUILTINUNIT_molecule" hasOnlySubstanceUnits="true"/>

<species id="mw0fc387f1_a053_4273_b301_eee7fd6053ee" name="FOXO1/3" compartment="mwc0549e25_5531_4b14_9196_afc5b3c40236" initialAmount="35928" substanceUnits="MWBUILTINUNIT_molecule" hasOnlySubstanceUnits="true"/>

<species id="mw17ab26e6_2cae_4b09_bd9b_d7b685bd9c73" name="IGF-R-C" compartment="mwc0549e25_5531_4b14_9196_afc5b3c40236" initialAmount="980" substanceUnits="MWBUILTINUNIT_molecule" hasOnlySubstanceUnits="true"/>

<species id="mwdb30ae98_436d_48b0_b344_8c393d0a73fd" name="IRS-1" compartment="mwc0549e25_5531_4b14_9196_afc5b3c40236" initialAmount="1772" substanceUnits="MWBUILTINUNIT_molecule" hasOnlySubstanceUnits="true"/>

<species id="mwde736097_4b47_42d4_bf63_48c2af8ebf99" name="PIP3" compartment="mwc0549e25_5531_4b14_9196_afc5b3c40236" initialAmount="11460" substanceUnits="MWBUILTINUNIT_molecule" hasOnlySubstanceUnits="true"/>

<species id="mw6155c54e_4a7e_4856_a4a9_1fcf68bf7b12" name="AKT P" compartment="mwc0549e25_5531_4b14_9196_afc5b3c40236" initialAmount="10000" substanceUnits="MWBUILTINUNIT_molecule" hasOnlySubstanceUnits="true"/>

<species id="mwf94121d8_c0d7_4448_8774_638eff8c2312" name="AKT UP" compartment="mwc0549e25_5531_4b14_9196_afc5b3c40236" initialAmount="100" substanceUnits="MWBUILTINUNIT_molecule" hasOnlySubstanceUnits="true" constant="true"/>

<species id="mw725cdfad_dc30_46d0_a778_10f361a8d4f2" name="IL-6" compartment="mwc0549e25_5531_4b14_9196_afc5b3c40236" initialAmount="35900" substanceUnits="MWBUILTINUNIT_molecule" hasOnlySubstanceUnits="true"/>

<species id="mw7e73ca07_cacb_437d_9f9e_addbaa62d69d" name="STAT3 D" compartment="mw4fe466a8_8f95_4dde_8a8b_18d4b7ad73b0" initialAmount="4000" substanceUnits="MWBUILTINUNIT_molecule" hasOnlySubstanceUnits="true"/>

<species id="mwac4fcd3b_4c80_42a8_9264_135e75f1d961" name="MuRF1" compartment="mw4fe466a8_8f95_4dde_8a8b_18d4b7ad73b0" initialAmount="120394" substanceUnits="MWBUILTINUNIT_molecule" hasOnlySubstanceUnits="true"/>

<species id="mwcbb05676_3442_49a8_b0a0_12f6e2897452" name="C/EBPD" compartment="mw4fe466a8_8f95_4dde_8a8b_18d4b7ad73b0" initialAmount="4000" substanceUnits="MWBUILTINUNIT_molecule" hasOnlySubstanceUnits="true"/>

<species id="mw22afa793_c236_4f05_8001_f721f2fcf3ac" name="NFKB" compartment="mw4fe466a8_8f95_4dde_8a8b_18d4b7ad73b0" initialAmount="4398" substanceUnits="MWBUILTINUNIT_molecule" hasOnlySubstanceUnits="true"/>

<species id="mwf2af60bf_deba_473f_8c43_4d7a8ffcefde" name="SOCS1/3" compartment="mw4fe466a8_8f95_4dde_8a8b_18d4b7ad73b0" initialAmount="4398" substanceUnits="MWBUILTINUNIT_molecule" hasOnlySubstanceUnits="true"/>

<species id="mw4e7c3e0a_6e14_4bb6_b846_b587ea107273" name="Myostatin" compartment="mw4fe466a8_8f95_4dde_8a8b_18d4b7ad73b0" initialAmount="4998" substanceUnits="MWBUILTINUNIT_molecule" hasOnlySubstanceUnits="true"/>

<species id="mwa5ec613a_4816_4904_9b5e_7e9b01695b91" name="ERK1/2" compartment="mw4fe466a8_8f95_4dde_8a8b_18d4b7ad73b0" initialAmount="6955" substanceUnits="MWBUILTINUNIT_molecule" hasOnlySubstanceUnits="true"/>

<species id="mw36823663_83dc_47b1_ade9_865411917d6d" name="DRP1" compartment="mw4fe466a8_8f95_4dde_8a8b_18d4b7ad73b0" initialAmount="9000" substanceUnits="MWBUILTINUNIT_molecule" hasOnlySubstanceUnits="true"/>

<species id="mw0e810310_40ad_4cfe_ad6f_889093f29968" name="FIS1" compartment="mw4fe466a8_8f95_4dde_8a8b_18d4b7ad73b0" initialAmount="19000" substanceUnits="MWBUILTINUNIT_molecule" hasOnlySubstanceUnits="true"/>

<species id="mwaa8adfd3_ef45_4b3d_b1c4_ee7c5364ad9b" name="SMAD2/3-4-C" compartment="mw4fe466a8_8f95_4dde_8a8b_18d4b7ad73b0" initialAmount="31864" substanceUnits="MWBUILTINUNIT_molecule" hasOnlySubstanceUnits="true"/>

<species id="mw9062efc2_a15b_4f4e_9f9d_0695aaa0f508" name="nfkb" compartment="mw4fe466a8_8f95_4dde_8a8b_18d4b7ad73b0" initialAmount="35000" substanceUnits="MWBUILTINUNIT_molecule" hasOnlySubstanceUnits="true"/>

<species id="mwca140588_6307_4ea5_9fb9_0f34bb654599" name="FOXO1/3" compartment="mw4fe466a8_8f95_4dde_8a8b_18d4b7ad73b0" initialAmount="35998" substanceUnits="MWBUILTINUNIT_molecule" hasOnlySubstanceUnits="true"/>

<species id="mw5f4cb81e_f578_43ca_9dbf_6f68315c839e" name="IL-6" compartment="mw4fe466a8_8f95_4dde_8a8b_18d4b7ad73b0" initialAmount="35920" substanceUnits="MWBUILTINUNIT_molecule" hasOnlySubstanceUnits="true"/>

<species id="mwa8bb8232_78cc_4d1b_bc42_a085dfd0941b" name="Foxo1/3" compartment="mw4fe466a8_8f95_4dde_8a8b_18d4b7ad73b0" initialAmount="45000" substanceUnits="MWBUILTINUNIT_molecule" hasOnlySubstanceUnits="true"/>

<species id="mwd994b0ae_580e_4bc9_9dc2_7012c5099e93" name="Atrogin-1" compartment="mw4fe466a8_8f95_4dde_8a8b_18d4b7ad73b0" initialAmount="120394" substanceUnits="MWBUILTINUNIT_molecule" hasOnlySubstanceUnits="true"/>

</listOfSpecies>

<listOfReactions>

<reaction id="mwd0186e54_2e0b_471e_bfe1_0f823c77bfd6" name="reaction_1" reversible="false" fast="false">

<listOfReactants>

<speciesReference species="mwea1c0c15_2b67_46fb_929b_7c1267ad9963"/>

</listOfReactants>

<listOfProducts>

<speciesReference species="mw37536c65_bf58_4c51_a2ee_d0100642163f"/>

</listOfProducts>

<kineticLaw>

<math xmlns="http://www.w3.org/1998/Math/MathML">

<apply>

<times/>

<ci> mw8abad9a2_3d38_42db_b89b_547f4caae6c8 </ci>

<ci> mwea1c0c15_2b67_46fb_929b_7c1267ad9963 </ci>

</apply>

</math>

<listOfParameters>

<parameter id="mw8abad9a2_3d38_42db_b89b_547f4caae6c8" name="kForward1" value="1.01e-009" units="MWDERIVEDUNIT_1__second"/>

</listOfParameters>

</kineticLaw>

</reaction>

<reaction id="mwb2e9ce16_7988_44cc_a2bb_648d54340f6f" name="reaction_2" reversible="false" fast="false">

<listOfReactants>

<speciesReference species="mw37536c65_bf58_4c51_a2ee_d0100642163f"/>

</listOfReactants>

<listOfProducts>

<speciesReference species="mw154f7171_cdd5_408d_b100_5d2d4faa8c9b"/>

</listOfProducts>

<kineticLaw>

<math xmlns="http://www.w3.org/1998/Math/MathML">

<apply>

<divide/>

<apply>

<times/>

<ci> mw74b6f970_0666_4235_82cc_38964e650a40 </ci>

<ci> mw37536c65_bf58_4c51_a2ee_d0100642163f </ci>

</apply>

<apply>

<plus/>

<ci> mwd0606be2_538e_46af_8b60_1a103772a337 </ci>

<ci> mw37536c65_bf58_4c51_a2ee_d0100642163f </ci>

</apply>

</apply>

</math>

<listOfParameters>

<parameter id="mw74b6f970_0666_4235_82cc_38964e650a40" name="Vm1" value="16" units="MWDERIVEDUNIT_molecule__second"/>

<parameter id="mwd0606be2_538e_46af_8b60_1a103772a337" name="Km1" value="8" units="MWBUILTINUNIT_molecule"/>

</listOfParameters>

</kineticLaw>

</reaction>

<reaction id="mwdc31f938_c2a4_40f1_90e4_a17b4a7084bd" name="reaction_3" reversible="false" fast="false">

<listOfReactants>

<speciesReference species="mw154f7171_cdd5_408d_b100_5d2d4faa8c9b"/>

</listOfReactants>

<listOfProducts>

<speciesReference species="mw486ef628_30a7_422a_b579_d3aa2c3f2fd4"/>

<speciesReference species="mwb67146fa_d038_4406_920d_414761f272e0"/>

<speciesReference species="mw16a20824_e3b6_4f7b_aa76_6a31fe8cbb0c"/>

</listOfProducts>

<kineticLaw>

<math xmlns="http://www.w3.org/1998/Math/MathML">

<apply>

<divide/>

<apply>

<times/>

<ci> mw4734b830_a297_4e89_be6e_47ef9de0c0b0 </ci>

<ci> mw154f7171_cdd5_408d_b100_5d2d4faa8c9b </ci>

</apply>

<apply>

<plus/>

<ci> mwaedfb6e8_aca0_4141_8087_5ba76eb8588c </ci>

<ci> mw154f7171_cdd5_408d_b100_5d2d4faa8c9b </ci>

</apply>

</apply>

</math>

<listOfParameters>

<parameter id="mw4734b830_a297_4e89_be6e_47ef9de0c0b0" name="Vm1" value="22" units="MWDERIVEDUNIT_molecule__second"/>

<parameter id="mwaedfb6e8_aca0_4141_8087_5ba76eb8588c" name="Km1" value="11" units="MWBUILTINUNIT_molecule"/>

</listOfParameters>

</kineticLaw>

</reaction>

<reaction id="mw3a97795c_9f49_4a82_8e87_7f8f2437b599" name="reaction_4" reversible="false" fast="false">

<listOfReactants>

<speciesReference species="mwb67146fa_d038_4406_920d_414761f272e0"/>

</listOfReactants>

<listOfProducts>

<speciesReference species="mwdf1f48ca_5474_4a18_a9ac_accc2ea8d515"/>

</listOfProducts>

<kineticLaw>

<math xmlns="http://www.w3.org/1998/Math/MathML">

<apply>

<divide/>

<apply>

<times/>

<ci> mw656f917a_8cfc_44de_9110_fd69c3b5105f </ci>

<ci> mwb67146fa_d038_4406_920d_414761f272e0 </ci>

</apply>

<apply>

<plus/>

<ci> mw24b71962_c4f4_49d9_bcd2_798d24d7e8d1 </ci>

<ci> mwb67146fa_d038_4406_920d_414761f272e0 </ci>

</apply>

</apply>

</math>

<listOfParameters>

<parameter id="mw656f917a_8cfc_44de_9110_fd69c3b5105f" name="Vm1" value="32" units="MWDERIVEDUNIT_molecule__second"/>

<parameter id="mw24b71962_c4f4_49d9_bcd2_798d24d7e8d1" name="Km1" value="16" units="MWBUILTINUNIT_molecule"/>

</listOfParameters>

</kineticLaw>

</reaction>

<reaction id="mw07c1bbd2_c4f6_43d9_b161_fe50f9244ed4" name="reaction_5" reversible="false" fast="false">

<listOfReactants>

<speciesReference species="mwdf1f48ca_5474_4a18_a9ac_accc2ea8d515"/>

</listOfReactants>

<listOfProducts>

<speciesReference species="mw7e73ca07_cacb_437d_9f9e_addbaa62d69d"/>

</listOfProducts>

<kineticLaw>

<math xmlns="http://www.w3.org/1998/Math/MathML">

<apply>

<times/>

<ci> mw39a4806e_be94_4556_8165_9ebe8e3ed144 </ci>

<ci> mwdf1f48ca_5474_4a18_a9ac_accc2ea8d515 </ci>

</apply>

</math>

<listOfParameters>

<parameter id="mw39a4806e_be94_4556_8165_9ebe8e3ed144" name="kForward1" value="0.01" units="MWDERIVEDUNIT_1__second"/>

</listOfParameters>

</kineticLaw>

</reaction>

<reaction id="mwebb3052d_d66c_436b_8a29_224d9cef78b6" name="reaction_6" reversible="false" fast="false">

<listOfReactants>

<speciesReference species="mw7e73ca07_cacb_437d_9f9e_addbaa62d69d"/>

</listOfReactants>

<listOfProducts>

<speciesReference species="mwac4fcd3b_4c80_42a8_9264_135e75f1d961"/>

<speciesReference species="mw22afa793_c236_4f05_8001_f721f2fcf3ac"/>

<speciesReference species="mwcbb05676_3442_49a8_b0a0_12f6e2897452"/>

<speciesReference species="mwf2af60bf_deba_473f_8c43_4d7a8ffcefde"/>

<speciesReference species="mwd994b0ae_580e_4bc9_9dc2_7012c5099e93"/>

</listOfProducts>

<kineticLaw>

<math xmlns="http://www.w3.org/1998/Math/MathML">

<apply>

<divide/>

<apply>

<times/>

<ci> mwf673bfec_ff95_40f7_a718_f63564e1a494 </ci>

<apply>

<power/>

<ci> mw7e73ca07_cacb_437d_9f9e_addbaa62d69d </ci>

<ci> mw60b6949e_e1ea_4bcf_9d40_db3b394451a3 </ci>

</apply>

</apply>

<apply>

<plus/>

<ci> mw00114fbe_9149_4758_8044_6a880dde6d1e </ci>

<apply>

<power/>

<ci> mw7e73ca07_cacb_437d_9f9e_addbaa62d69d </ci>

<ci> mw60b6949e_e1ea_4bcf_9d40_db3b394451a3 </ci>

</apply>

</apply>

</apply>

</math>

<listOfParameters>

<parameter id="mwf673bfec_ff95_40f7_a718_f63564e1a494" name="Vm1" value="20" units="MWDERIVEDUNIT_molecule__second"/>

<parameter id="mw60b6949e_e1ea_4bcf_9d40_db3b394451a3" name="n1" value="1"/>

<parameter id="mw00114fbe_9149_4758_8044_6a880dde6d1e" name="Kp1" value="1" units="MWBUILTINUNIT_molecule"/>

</listOfParameters>

</kineticLaw>

</reaction>

<reaction id="mw9d52f789_45f4_4afc_b844_212bd8dba0b4" name="reaction_7" reversible="false" fast="false">

<listOfReactants>

<speciesReference species="mwcbb05676_3442_49a8_b0a0_12f6e2897452"/>

</listOfReactants>

<listOfProducts>

<speciesReference species="mw4e7c3e0a_6e14_4bb6_b846_b587ea107273"/>

</listOfProducts>

<kineticLaw>

<math xmlns="http://www.w3.org/1998/Math/MathML">

<apply>

<divide/>

<apply>

<times/>

<ci> mw274439db_1d05_4717_afc7_9412cb1dcd00 </ci>

<apply>

<power/>

<ci> mwcbb05676_3442_49a8_b0a0_12f6e2897452 </ci>

<ci> mw163bbabb_9d26_4279_84c1_83877ed669e3 </ci>

</apply>

</apply>

<apply>

<plus/>

<ci> mwbc6b510b_ef4c_4e66_8de9_68dad77b3699 </ci>

<apply>

<power/>

<ci> mwcbb05676_3442_49a8_b0a0_12f6e2897452 </ci>

<ci> mw163bbabb_9d26_4279_84c1_83877ed669e3 </ci>

</apply>

</apply>

</apply>

</math>

<listOfParameters>

<parameter id="mw274439db_1d05_4717_afc7_9412cb1dcd00" name="Vm1" value="30" units="MWDERIVEDUNIT_molecule__second"/>

<parameter id="mwbc6b510b_ef4c_4e66_8de9_68dad77b3699" name="Kp1" value="1" units="MWBUILTINUNIT_molecule"/>

<parameter id="mw163bbabb_9d26_4279_84c1_83877ed669e3" name="n1" value="1"/>

</listOfParameters>

</kineticLaw>

</reaction>

<reaction id="mw87e1051b_242e_4bf4_9cca_466f1d844b3a" name="reaction_8" reversible="false" fast="false">

<listOfReactants>

<speciesReference species="mw486ef628_30a7_422a_b579_d3aa2c3f2fd4"/>

</listOfReactants>

<listOfProducts>

<speciesReference species="mwbfbf065a_5322_4a5e_8f93_6cd8ffb41ea7"/>

</listOfProducts>

<kineticLaw>

<math xmlns="http://www.w3.org/1998/Math/MathML">

<apply>

<divide/>

<apply>

<times/>

<ci> mw1f1420a6_b48b_4960_89e0_10aa54b1f98f </ci>

<ci> mw486ef628_30a7_422a_b579_d3aa2c3f2fd4 </ci>

</apply>

<apply>

<plus/>

<ci> mw1343c630_87df_4787_81a8_768af52979b6 </ci>

<ci> mw486ef628_30a7_422a_b579_d3aa2c3f2fd4 </ci>

</apply>

</apply>

</math>

<listOfParameters>

<parameter id="mw1f1420a6_b48b_4960_89e0_10aa54b1f98f" name="Vm1" value="16" units="MWDERIVEDUNIT_molecule__second"/>

<parameter id="mw1343c630_87df_4787_81a8_768af52979b6" name="Km1" value="8" units="MWBUILTINUNIT_molecule"/>

</listOfParameters>

</kineticLaw>

</reaction>

<reaction id="mwa135e220_5f4d_4d47_8329_b988deb19778" name="reaction_9" reversible="false" fast="false">

<listOfReactants>

<speciesReference species="mwbfbf065a_5322_4a5e_8f93_6cd8ffb41ea7"/>

</listOfReactants>

<listOfProducts>

<speciesReference species="mw89b78fe1_5fdd_4290_8d1e_0cea4ac30f64"/>

</listOfProducts>

<kineticLaw>

<math xmlns="http://www.w3.org/1998/Math/MathML">

<apply>

<divide/>

<apply>

<times/>

<ci> mw0d4ddd63_07ec_49d6_a092_f007d9d9eed2 </ci>

<ci> mwbfbf065a_5322_4a5e_8f93_6cd8ffb41ea7 </ci>

</apply>

<apply>

<plus/>

<ci> mwf7b1de17_ac91_4bf7_9dc5_6aa2bb5bec9e </ci>

<ci> mwbfbf065a_5322_4a5e_8f93_6cd8ffb41ea7 </ci>

</apply>

</apply>

</math>

<listOfParameters>

<parameter id="mw0d4ddd63_07ec_49d6_a092_f007d9d9eed2" name="Vm1" value="26" units="MWDERIVEDUNIT_molecule__second"/>

<parameter id="mwf7b1de17_ac91_4bf7_9dc5_6aa2bb5bec9e" name="Km1" value="13" units="MWBUILTINUNIT_molecule"/>

</listOfParameters>

</kineticLaw>

</reaction>

<reaction id="mw03339b00_2a71_46e7_b458_4436f17e8256" name="reaction_10" reversible="false" fast="false">

<listOfReactants>

<speciesReference species="mw89b78fe1_5fdd_4290_8d1e_0cea4ac30f64"/>

</listOfReactants>

<listOfProducts>

<speciesReference species="mwf9e54da4_564e_460f_aee4_28eb334e5fe9"/>

</listOfProducts>

<kineticLaw>

<math xmlns="http://www.w3.org/1998/Math/MathML">

<apply>

<divide/>

<apply>

<times/>

<ci> mw00c02529_63f4_4e3a_8ec3_3e02df671ba2 </ci>

<ci> mw89b78fe1_5fdd_4290_8d1e_0cea4ac30f64 </ci>

</apply>

<apply>

<plus/>

<ci> mwc40b0e6e_cf57_4da5_8d86_1c0e80e29c25 </ci>

<ci> mw89b78fe1_5fdd_4290_8d1e_0cea4ac30f64 </ci>

</apply>

</apply>

</math>

<listOfParameters>

<parameter id="mw00c02529_63f4_4e3a_8ec3_3e02df671ba2" name="Vm1" value="32" units="MWDERIVEDUNIT_molecule__second"/>

<parameter id="mwc40b0e6e_cf57_4da5_8d86_1c0e80e29c25" name="Km1" value="16" units="MWBUILTINUNIT_molecule"/>

</listOfParameters>

</kineticLaw>

</reaction>

<reaction id="mw8604812e_3751_4c05_b84a_3888c31308f0" name="reaction_11" reversible="false" fast="false">

<listOfReactants>

<speciesReference species="mwf9e54da4_564e_460f_aee4_28eb334e5fe9"/>

</listOfReactants>

<listOfProducts>

<speciesReference species="mw88274cec_267a_4a84_9cc3_de3d0eaac1cb"/>

</listOfProducts>

<kineticLaw>

<math xmlns="http://www.w3.org/1998/Math/MathML">

<apply>

<divide/>

<apply>

<times/>

<ci> mw1a426b14_dec4_4a67_bf9e_4078962803d3 </ci>

<ci> mwf9e54da4_564e_460f_aee4_28eb334e5fe9 </ci>

</apply>

<apply>

<plus/>

<ci> mw86e9271d_73f7_4e8e_a3cd_994da6aac7cb </ci>

<ci> mwf9e54da4_564e_460f_aee4_28eb334e5fe9 </ci>

</apply>

</apply>

</math>

<listOfParameters>

<parameter id="mw1a426b14_dec4_4a67_bf9e_4078962803d3" name="Vm1" value="32" units="MWDERIVEDUNIT_molecule__second"/>

<parameter id="mw86e9271d_73f7_4e8e_a3cd_994da6aac7cb" name="Km1" value="16" units="MWBUILTINUNIT_molecule"/>

</listOfParameters>

</kineticLaw>

</reaction>

<reaction id="mw13ea02b0_b2a0_4520_bf78_bea4ae274e75" name="reaction_12" reversible="false" fast="false">

<listOfReactants>

<speciesReference species="mw88274cec_267a_4a84_9cc3_de3d0eaac1cb"/>

</listOfReactants>

<listOfProducts>

<speciesReference species="mwa5ec613a_4816_4904_9b5e_7e9b01695b91"/>

</listOfProducts>

<kineticLaw>

<math xmlns="http://www.w3.org/1998/Math/MathML">

<apply>

<times/>

<ci> mw444da69d_d358_41dc_ac4d_2525eb919f7f </ci>

<ci> mw88274cec_267a_4a84_9cc3_de3d0eaac1cb </ci>

</apply>

</math>

<listOfParameters>

<parameter id="mw444da69d_d358_41dc_ac4d_2525eb919f7f" name="kForward1" value="0.0099" units="MWDERIVEDUNIT_1__second"/>

</listOfParameters>

</kineticLaw>

</reaction>

<reaction id="mw3280f9f3_2a1d_439f_936c_cd0c5a3c5ceb" name="reaction_14" reversible="false" fast="false">

<listOfReactants>

<speciesReference species="mw0e810310_40ad_4cfe_ad6f_889093f29968"/>

</listOfReactants>

<listOfProducts>

<speciesReference species="mw759859f4_f3b3_48e7_bf82_740506b598b0"/>

</listOfProducts>

<kineticLaw>

<math xmlns="http://www.w3.org/1998/Math/MathML">

<apply>

<times/>

<ci> mwd4a165bb_7c04_4fd1_ad01_eddaa5f3f064 </ci>

<ci> mw0e810310_40ad_4cfe_ad6f_889093f29968 </ci>

</apply>

</math>

<listOfParameters>

<parameter id="mwd4a165bb_7c04_4fd1_ad01_eddaa5f3f064" name="kForward1" value="0.02055145127" units="MWDERIVEDUNIT_1__second"/>

</listOfParameters>

</kineticLaw>

</reaction>

<reaction id="mw156cc2e4_f909_462e_bb95_cbe1c5abf10c" name="reaction_16" reversible="false" fast="false">

<listOfReactants>

<speciesReference species="mw759859f4_f3b3_48e7_bf82_740506b598b0"/>

</listOfReactants>

<listOfProducts>

<speciesReference species="mw34aeb325_8500_4f88_8835_0d633dbea428"/>

</listOfProducts>

<kineticLaw>

<math xmlns="http://www.w3.org/1998/Math/MathML">

<apply>

<divide/>

<apply>

<times/>

<ci> mw36546c27_e4e4_47f5_96e6_5dd790ab9d64 </ci>

<ci> mw759859f4_f3b3_48e7_bf82_740506b598b0 </ci>

</apply>

<apply>

<plus/>

<ci> mwe260611b_935e_47f7_9166_19e1c3699592 </ci>

<ci> mw759859f4_f3b3_48e7_bf82_740506b598b0 </ci>

</apply>

</apply>

</math>

<listOfParameters>

<parameter id="mw36546c27_e4e4_47f5_96e6_5dd790ab9d64" name="Vm1" value="68" units="MWDERIVEDUNIT_molecule__second"/>

<parameter id="mwe260611b_935e_47f7_9166_19e1c3699592" name="Km1" value="34" units="MWBUILTINUNIT_molecule"/>

</listOfParameters>

</kineticLaw>

</reaction>

<reaction id="mw2c2f07d8_273f_4dc0_a0ff_7b7b2135a25d" name="reaction_17" reversible="false" fast="false">

<listOfReactants>

<speciesReference species="mweb909918_2d84_489e_a6e4_a8b51c6b2040"/>

</listOfReactants>

<listOfProducts>

<speciesReference species="mw34aeb325_8500_4f88_8835_0d633dbea428"/>

</listOfProducts>

<kineticLaw>

<math xmlns="http://www.w3.org/1998/Math/MathML">

<apply>

<divide/>

<apply>

<times/>

<ci> mw353752f9_83c7_4315_835f_6406e67b300c </ci>

<ci> mweb909918_2d84_489e_a6e4_a8b51c6b2040 </ci>

</apply>

<apply>

<plus/>

<ci> mwb53f421b_a79e_4372_a3cd_f54d0e4a893b </ci>

<ci> mweb909918_2d84_489e_a6e4_a8b51c6b2040 </ci>

</apply>

</apply>

</math>

<listOfParameters>

<parameter id="mw353752f9_83c7_4315_835f_6406e67b300c" name="Vm1" value="84" units="MWDERIVEDUNIT_molecule__second"/>

<parameter id="mwb53f421b_a79e_4372_a3cd_f54d0e4a893b" name="Km1" value="42" units="MWBUILTINUNIT_molecule"/>

</listOfParameters>

</kineticLaw>

</reaction>

<reaction id="mwc7df3bbe_5886_4ed7_8459_6d2d7375528c" name="reaction_18" reversible="false" fast="false">

<listOfReactants>

<speciesReference species="mw34aeb325_8500_4f88_8835_0d633dbea428"/>

</listOfReactants>

<listOfProducts>

<speciesReference species="mwfd54e35c_b0e8_4374_b666_981e29d936c6"/>

</listOfProducts>

<kineticLaw>

<math xmlns="http://www.w3.org/1998/Math/MathML">

<apply>

<divide/>

<apply>

<times/>

<ci> mwfef10d0c_6dfb_4a8d_b0be_b8f3a18107e4 </ci>

<ci> mw34aeb325_8500_4f88_8835_0d633dbea428 </ci>

</apply>

<apply>

<plus/>

<ci> mw75d3e2cd_cfb2_4156_bedb_eb501f765367 </ci>

<ci> mw34aeb325_8500_4f88_8835_0d633dbea428 </ci>

</apply>

</apply>

</math>

<listOfParameters>

<parameter id="mwfef10d0c_6dfb_4a8d_b0be_b8f3a18107e4" name="Vm1" value="220" units="MWDERIVEDUNIT_molecule__second"/>

<parameter id="mw75d3e2cd_cfb2_4156_bedb_eb501f765367" name="Km1" value="110" units="MWBUILTINUNIT_molecule"/>

</listOfParameters>

</kineticLaw>

</reaction>

<reaction id="mw492004d3_fd1c_4deb_91af_49ffc752bfd3" name="reaction_19" reversible="false" fast="false">

<listOfReactants>

<speciesReference species="mw4e7c3e0a_6e14_4bb6_b846_b587ea107273"/>

</listOfReactants>

<listOfProducts>

<speciesReference species="mw1e8b346c_6576_495e_9457_539c8f2a3023"/>

</listOfProducts>

<kineticLaw>

<math xmlns="http://www.w3.org/1998/Math/MathML">

<apply>

<times/>

<ci> mw81ac7de3_cb6e_4e5e_be81_e2a0d8a99774 </ci>

<ci> mw4e7c3e0a_6e14_4bb6_b846_b587ea107273 </ci>

</apply>

</math>

<listOfParameters>

<parameter id="mw81ac7de3_cb6e_4e5e_be81_e2a0d8a99774" name="kForward1" value="0.04" units="MWDERIVEDUNIT_1__second"/>

</listOfParameters>

</kineticLaw>

</reaction>

<reaction id="mwcb2c9283_b11e_4fdb_9e15_f9ec55d878c4" name="reaction_20" reversible="false" fast="false">

<listOfReactants>

<speciesReference species="mwfd54e35c_b0e8_4374_b666_981e29d936c6"/>

</listOfReactants>

<listOfProducts>

<speciesReference species="mwf3f1e954_4fa7_4d92_b277_2062ac96263a"/>

<speciesReference species="mw26bfd5c7_b3a6_4615_8b41_7ac6a7c78c1b"/>

<speciesReference species="mw1e8b346c_6576_495e_9457_539c8f2a3023"/>

</listOfProducts>

<kineticLaw>

<math xmlns="http://www.w3.org/1998/Math/MathML">

<apply>

<divide/>

<apply>

<times/>

<ci> mwd8e62ed9_15d2_4c5d_a98f_475f2865b3cc </ci>

<ci> mwfd54e35c_b0e8_4374_b666_981e29d936c6 </ci>

</apply>

<apply>

<plus/>

<ci> mwbd764b09_6160_44a5_8ebd_39b30bd1d321 </ci>

<ci> mwfd54e35c_b0e8_4374_b666_981e29d936c6 </ci>

</apply>

</apply>

</math>

<listOfParameters>

<parameter id="mwd8e62ed9_15d2_4c5d_a98f_475f2865b3cc" name="Vm1" value="58" units="MWDERIVEDUNIT_molecule__second"/>

<parameter id="mwbd764b09_6160_44a5_8ebd_39b30bd1d321" name="Km1" value="29" units="MWBUILTINUNIT_molecule"/>

</listOfParameters>

</kineticLaw>

</reaction>

<reaction id="mw4b2b6b46_133e_4af7_9c2d_77302dccb9af" name="reaction_21" reversible="false" fast="false">

<listOfReactants>

<speciesReference species="mw1e8b346c_6576_495e_9457_539c8f2a3023"/>

</listOfReactants>

<listOfProducts>

<speciesReference species="mw8b46f320_13f4_457c_bd6f_e2cea1cd0e03"/>

</listOfProducts>

<kineticLaw>

<math xmlns="http://www.w3.org/1998/Math/MathML">

<apply>

<times/>

<ci> mwd34f9dea_2d43_475c_8a61_2bad8c3e6c49 </ci>

<ci> mw1e8b346c_6576_495e_9457_539c8f2a3023 </ci>

</apply>

</math>

<listOfParameters>

<parameter id="mwd34f9dea_2d43_475c_8a61_2bad8c3e6c49" name="kForward1" value="0.02" units="MWDERIVEDUNIT_1__second"/>

</listOfParameters>

</kineticLaw>

</reaction>

<reaction id="mwa57535c6_e21c_4233_a14d_4ee6423e76c9" name="reaction_22" reversible="false" fast="false">

<listOfReactants>

<speciesReference species="mw8b46f320_13f4_457c_bd6f_e2cea1cd0e03"/>

</listOfReactants>

<listOfProducts>

<speciesReference species="mw0fb4af26_aaa9_46cf_820f_a01414045446"/>

</listOfProducts>

<kineticLaw>

<math xmlns="http://www.w3.org/1998/Math/MathML">

<apply>

<times/>

<ci> mweceea25d_b6b0_4798_9401_bdd117ee781d </ci>

<ci> mw8b46f320_13f4_457c_bd6f_e2cea1cd0e03 </ci>

</apply>

</math>

<listOfParameters>

<parameter id="mweceea25d_b6b0_4798_9401_bdd117ee781d" name="kForward1" value="1.01e-009" units="MWDERIVEDUNIT_1__second"/>

</listOfParameters>

</kineticLaw>

</reaction>

<reaction id="mw596311ec_0250_448d_b33c_c6f9b0dd156a" name="reaction_23" reversible="false" fast="false">

<listOfReactants>

<speciesReference species="mw0fb4af26_aaa9_46cf_820f_a01414045446"/>

</listOfReactants>

<listOfProducts>

<speciesReference species="mwab852ab4_c2c5_4647_a2ac_b14e2642d48b"/>

</listOfProducts>

<kineticLaw>

<math xmlns="http://www.w3.org/1998/Math/MathML">

<apply>

<divide/>

<apply>

<times/>

<ci> mwd787c6d3_8a11_493d_b453_64c38b0c164f </ci>

<ci> mw0fb4af26_aaa9_46cf_820f_a01414045446 </ci>

</apply>

<apply>

<plus/>

<ci> mwa303b8d3_61d7_44e6_84f1_4a5e15bc78fb </ci>

<ci> mw0fb4af26_aaa9_46cf_820f_a01414045446 </ci>

</apply>

</apply>

</math>

<listOfParameters>

<parameter id="mwd787c6d3_8a11_493d_b453_64c38b0c164f" name="Vm1" value="400" units="MWDERIVEDUNIT_molecule__second"/>

<parameter id="mwa303b8d3_61d7_44e6_84f1_4a5e15bc78fb" name="Km1" value="200" units="MWBUILTINUNIT_molecule"/>

</listOfParameters>

</kineticLaw>

</reaction>

<reaction id="mw19e3b2e7_c5b1_4ee3_85a6_41d970cbd640" name="reaction_24" reversible="false" fast="false">

<listOfReactants>

<speciesReference species="mwab852ab4_c2c5_4647_a2ac_b14e2642d48b"/>

</listOfReactants>

<listOfProducts>

<speciesReference species="mw3d1df92d_2bbf_45fa_8039_0f7a0a668b47"/>

</listOfProducts>

<kineticLaw>

<math xmlns="http://www.w3.org/1998/Math/MathML">

<apply>

<times/>

<ci> mwfdea53f5_e775_44fc_85ec_07e0be94b63e </ci>

<ci> mwab852ab4_c2c5_4647_a2ac_b14e2642d48b </ci>

</apply>

</math>

<listOfParameters>

<parameter id="mwfdea53f5_e775_44fc_85ec_07e0be94b63e" name="kForward1" value="0.0003050922" units="MWDERIVEDUNIT_1__second"/>

</listOfParameters>

</kineticLaw>

</reaction>

<reaction id="mw3351dced_8e69_406e_8f70_8e06a4e10d81" name="reaction_25" reversible="false" fast="false">

<listOfReactants>

<speciesReference species="mw3d1df92d_2bbf_45fa_8039_0f7a0a668b47"/>

</listOfReactants>

<listOfProducts>

<speciesReference species="mwaa8adfd3_ef45_4b3d_b1c4_ee7c5364ad9b"/>

</listOfProducts>

<kineticLaw>

<math xmlns="http://www.w3.org/1998/Math/MathML">

<apply>

<times/>

<ci> mw098f57f5_312c_40a2_9ca3_cc16e5e68c28 </ci>

<ci> mw3d1df92d_2bbf_45fa_8039_0f7a0a668b47 </ci>

</apply>

</math>

<listOfParameters>

<parameter id="mw098f57f5_312c_40a2_9ca3_cc16e5e68c28" name="kForward1" value="0.0075" units="MWDERIVEDUNIT_1__second"/>

</listOfParameters>

</kineticLaw>

</reaction>

<reaction id="mwff638065_23ba_4fe6_99d7_242a37c83df1" name="reaction_26" reversible="false" fast="false">

<listOfReactants>

<speciesReference species="mwaa8adfd3_ef45_4b3d_b1c4_ee7c5364ad9b"/>

</listOfReactants>

<listOfProducts>

<speciesReference species="mwac4fcd3b_4c80_42a8_9264_135e75f1d961"/>

<speciesReference species="mwd994b0ae_580e_4bc9_9dc2_7012c5099e93"/>

</listOfProducts>

<kineticLaw>

<math xmlns="http://www.w3.org/1998/Math/MathML">

<apply>

<divide/>

<apply>

<times/>

<ci> mw19f7955a_f0ba_4f9d_a335_2b6c0e67ae22 </ci>

<apply>

<power/>

<ci> mwaa8adfd3_ef45_4b3d_b1c4_ee7c5364ad9b </ci>

<ci> mw02cd6d3b_fa21_4001_9fd1_cc59c2e0f6cf </ci>

</apply>

</apply>

<apply>

<plus/>

<ci> mwb0e2cb40_3f94_4760_abef_063597f7ae07 </ci>

<apply>

<power/>

<ci> mwaa8adfd3_ef45_4b3d_b1c4_ee7c5364ad9b </ci>

<ci> mw02cd6d3b_fa21_4001_9fd1_cc59c2e0f6cf </ci>

</apply>

</apply>

</apply>

</math>

<listOfParameters>

<parameter id="mw19f7955a_f0ba_4f9d_a335_2b6c0e67ae22" name="Vm1" value="382" units="MWDERIVEDUNIT_molecule__second"/>

<parameter id="mw02cd6d3b_fa21_4001_9fd1_cc59c2e0f6cf" name="n1" value="1"/>

<parameter id="mwb0e2cb40_3f94_4760_abef_063597f7ae07" name="Kp1" value="1" units="MWBUILTINUNIT_molecule"/>

</listOfParameters>

</kineticLaw>

</reaction>

<reaction id="mwdc162459_8ec9_40f2_a51c_b89b6ff22812" name="reaction_27" reversible="false" fast="false">

<listOfReactants>

<speciesReference species="mwc0a9a42d_48d4_468f_bab9_574edacbe15a"/>

<speciesReference species="mwfbd3d650_d9b8_4fe3_9643_0a891d49c063"/>

</listOfReactants>

<listOfProducts>

<speciesReference species="mw2552df9f_943e_4f52_a55d_cf0e98b255c3"/>

</listOfProducts>

<kineticLaw>

<math xmlns="http://www.w3.org/1998/Math/MathML">

<apply>

<times/>

<ci> mwf55cc682_8d31_44fd_b44b_8e6f8b5df000 </ci>

<ci> mwc0a9a42d_48d4_468f_bab9_574edacbe15a </ci>

<ci> mwfbd3d650_d9b8_4fe3_9643_0a891d49c063 </ci>

</apply>

</math>

<listOfParameters>

<parameter id="mwf55cc682_8d31_44fd_b44b_8e6f8b5df000" name="kForward1" value="5e-006" units="MWDERIVEDUNIT_1__molecule_second"/>

</listOfParameters>

</kineticLaw>

</reaction>

<reaction id="mwff78b75a_324a_4194_9fe8_25374979ed18" name="reaction_28" reversible="false" fast="false">

<listOfReactants>

<speciesReference species="mw2552df9f_943e_4f52_a55d_cf0e98b255c3"/>

</listOfReactants>

<listOfProducts>

<speciesReference species="mwa847064b_ceba_41e5_b9c8_0a8f98dc781a"/>

</listOfProducts>

<kineticLaw>

<math xmlns="http://www.w3.org/1998/Math/MathML">

<apply>

<divide/>

<apply>

<times/>

<ci> mw97a63c3f_5d23_4f72_8df7_71f89bbf835b </ci>

<ci> mw2552df9f_943e_4f52_a55d_cf0e98b255c3 </ci>

</apply>

<apply>

<plus/>

<ci> mw6c37d8a6_6380_49e0_9d77_9fc79eb76c7a </ci>

<ci> mw2552df9f_943e_4f52_a55d_cf0e98b255c3 </ci>

</apply>

</apply>

</math>

<listOfParameters>

<parameter id="mw97a63c3f_5d23_4f72_8df7_71f89bbf835b" name="Vm1" value="18" units="MWDERIVEDUNIT_molecule__second"/>

<parameter id="mw6c37d8a6_6380_49e0_9d77_9fc79eb76c7a" name="Km1" value="9" units="MWBUILTINUNIT_molecule"/>

</listOfParameters>

</kineticLaw>

</reaction>

<reaction id="mw5116ad1f_3240_437e_b0cb_5e5d78b67056" name="reaction_29" reversible="false" fast="false">

<listOfReactants>

<speciesReference species="mwa847064b_ceba_41e5_b9c8_0a8f98dc781a"/>

</listOfReactants>

<listOfProducts>

<speciesReference species="mw26bfd5c7_b3a6_4615_8b41_7ac6a7c78c1b"/>

</listOfProducts>

<kineticLaw>

<math xmlns="http://www.w3.org/1998/Math/MathML">

<apply>

<divide/>

<apply>

<times/>

<ci> mw700376ff_08cc_495e_8939_31abeb60d8e4 </ci>

<ci> mwa847064b_ceba_41e5_b9c8_0a8f98dc781a </ci>

</apply>

<apply>

<plus/>

<ci> mwaa29854a_2355_4d3a_9117_80e71dd8f37f </ci>

<ci> mwa847064b_ceba_41e5_b9c8_0a8f98dc781a </ci>

</apply>

</apply>

</math>

<listOfParameters>

<parameter id="mw700376ff_08cc_495e_8939_31abeb60d8e4" name="Vm1" value="50" units="MWDERIVEDUNIT_molecule__second"/>

<parameter id="mwaa29854a_2355_4d3a_9117_80e71dd8f37f" name="Km1" value="25" units="MWBUILTINUNIT_molecule"/>

</listOfParameters>

</kineticLaw>

</reaction>

<reaction id="mwaab2d8a0_e550_4d6d_a116_cec9029d8248" name="reaction_30" reversible="false" fast="false">

<listOfReactants>

<speciesReference species="mw22afa793_c236_4f05_8001_f721f2fcf3ac"/>

</listOfReactants>

<listOfProducts>

<speciesReference species="mw26bfd5c7_b3a6_4615_8b41_7ac6a7c78c1b"/>

</listOfProducts>

<kineticLaw>

<math xmlns="http://www.w3.org/1998/Math/MathML">

<apply>

<times/>

<ci> mw6392c7bc_022e_4154_a47b_5ee7eedc3b82 </ci>

<ci> mw22afa793_c236_4f05_8001_f721f2fcf3ac </ci>

</apply>

</math>

<listOfParameters>

<parameter id="mw6392c7bc_022e_4154_a47b_5ee7eedc3b82" name="kForward1" value="0.009" units="MWDERIVEDUNIT_1__second"/>

</listOfParameters>

</kineticLaw>

</reaction>

<reaction id="mwd8bb7dc8_7a7f_4bd9_846f_857e612314f4" name="reaction_31" reversible="false" fast="false">

<listOfReactants>

<speciesReference species="mw26bfd5c7_b3a6_4615_8b41_7ac6a7c78c1b"/>

</listOfReactants>

<listOfProducts>

<speciesReference species="mw9062efc2_a15b_4f4e_9f9d_0695aaa0f508"/>

</listOfProducts>

<kineticLaw>

<math xmlns="http://www.w3.org/1998/Math/MathML">

<apply>

<times/>

<ci> mwd0da9606_a4be_4ce6_8972_f9ff7cc3ab45 </ci>

<ci> mw26bfd5c7_b3a6_4615_8b41_7ac6a7c78c1b </ci>

</apply>

</math>

<listOfParameters>

<parameter id="mwd0da9606_a4be_4ce6_8972_f9ff7cc3ab45" name="kForward1" value="0.009" units="MWDERIVEDUNIT_1__second"/>

</listOfParameters>

</kineticLaw>

</reaction>

<reaction id="mwa5e2b7c4_fd32_4de8_ae4d_bb40c3b0365e" name="reaction_32" reversible="false" fast="false">

<listOfReactants>

<speciesReference species="mw9062efc2_a15b_4f4e_9f9d_0695aaa0f508"/>

</listOfReactants>

<listOfProducts>

<speciesReference species="mwac4fcd3b_4c80_42a8_9264_135e75f1d961"/>

<speciesReference species="mwca140588_6307_4ea5_9fb9_0f34bb654599"/>

<speciesReference species="mw5f4cb81e_f578_43ca_9dbf_6f68315c839e"/>

<speciesReference species="mwd994b0ae_580e_4bc9_9dc2_7012c5099e93"/>

</listOfProducts>

<kineticLaw>

<math xmlns="http://www.w3.org/1998/Math/MathML">

<apply>

<divide/>

<apply>

<times/>

<ci> mwb5f309c4_49ea_4af0_8023_dac6d60d5517 </ci>

<apply>

<power/>

<ci> mw9062efc2_a15b_4f4e_9f9d_0695aaa0f508 </ci>

<ci> mwe11d4576_eed5_4df3_99c5_c927381cf303 </ci>

</apply>

</apply>

<apply>

<plus/>

<ci> mw69f6aebb_7d5f_408b_bf32_7e79998f3f5c </ci>

<apply>

<power/>

<ci> mw9062efc2_a15b_4f4e_9f9d_0695aaa0f508 </ci>

<ci> mwe11d4576_eed5_4df3_99c5_c927381cf303 </ci>

</apply>

</apply>

</apply>

</math>

<listOfParameters>

<parameter id="mwb5f309c4_49ea_4af0_8023_dac6d60d5517" name="Vm1" value="152" units="MWDERIVEDUNIT_molecule__second"/>

<parameter id="mwe11d4576_eed5_4df3_99c5_c927381cf303" name="n1" value="1"/>

<parameter id="mw69f6aebb_7d5f_408b_bf32_7e79998f3f5c" name="Kp1" value="1" units="MWBUILTINUNIT_molecule"/>

</listOfParameters>

</kineticLaw>

</reaction>

<reaction id="mw1d441c0d_cd5b_4a68_b92a_904fa4a03b91" name="reaction_33" reversible="false" fast="false">

<listOfReactants>

<speciesReference species="mwca140588_6307_4ea5_9fb9_0f34bb654599"/>

</listOfReactants>

<listOfProducts>

<speciesReference species="mw0fc387f1_a053_4273_b301_eee7fd6053ee"/>

</listOfProducts>

<kineticLaw>

<math xmlns="http://www.w3.org/1998/Math/MathML">

<apply>

<times/>

<ci> mw9d0230d6_c3c3_457a_98a3_c9314ddb7864 </ci>

<ci> mwca140588_6307_4ea5_9fb9_0f34bb654599 </ci>

</apply>

</math>

<listOfParameters>

<parameter id="mw9d0230d6_c3c3_457a_98a3_c9314ddb7864" name="kForward1" value="0.01" units="MWDERIVEDUNIT_1__second"/>

</listOfParameters>

</kineticLaw>

</reaction>

<reaction id="mw5bf4c469_4e46_4563_808c_2c6f4f82305a" name="reaction_34" reversible="false" fast="false">

<listOfReactants>

<speciesReference species="mwed30a3ca_aba6_4030_9bb8_0ad3fff03228"/>

<speciesReference species="mwee2376de_9c09_486e_af20_4eeb71df78d1"/>

</listOfReactants>

<listOfProducts>

<speciesReference species="mw17ab26e6_2cae_4b09_bd9b_d7b685bd9c73"/>

</listOfProducts>

<kineticLaw>

<math xmlns="http://www.w3.org/1998/Math/MathML">

<apply>

<times/>

<ci> mwa8ca766b_3ea8_40a6_a778_29364c1d3751 </ci>

<ci> mwed30a3ca_aba6_4030_9bb8_0ad3fff03228 </ci>

<ci> mwee2376de_9c09_486e_af20_4eeb71df78d1 </ci>

</apply>

</math>

<listOfParameters>

<parameter id="mwa8ca766b_3ea8_40a6_a778_29364c1d3751" name="kForward1" value="1e-005" units="MWDERIVEDUNIT_1__molecule_second"/>

</listOfParameters>

</kineticLaw>

</reaction>

<reaction id="mw9c849c34_5cf9_4247_bdfc_e98c0750d550" name="reaction_35" reversible="false" fast="false">

<listOfReactants>

<speciesReference species="mw17ab26e6_2cae_4b09_bd9b_d7b685bd9c73"/>

</listOfReactants>

<listOfProducts>

<speciesReference species="mwdb30ae98_436d_48b0_b344_8c393d0a73fd"/>

</listOfProducts>

<kineticLaw>

<math xmlns="http://www.w3.org/1998/Math/MathML">

<apply>

<divide/>

<apply>

<times/>

<ci> mw39afae15_65bb_4cf0_bfe5_1274e925f856 </ci>

<ci> mw17ab26e6_2cae_4b09_bd9b_d7b685bd9c73 </ci>

</apply>

<apply>

<plus/>

<ci> mwd57ac950_dffc_40d4_9bd9_d245e7e5088f </ci>

<ci> mw17ab26e6_2cae_4b09_bd9b_d7b685bd9c73 </ci>

</apply>

</apply>

</math>

<listOfParameters>

<parameter id="mw39afae15_65bb_4cf0_bfe5_1274e925f856" name="Vm1" value="30" units="MWDERIVEDUNIT_molecule__second"/>

<parameter id="mwd57ac950_dffc_40d4_9bd9_d245e7e5088f" name="Km1" value="15" units="MWBUILTINUNIT_molecule"/>

</listOfParameters>

</kineticLaw>

</reaction>

<reaction id="mw1fd4ae39_3217_480e_8bcc_a85e6ad83faf" name="reaction_36" reversible="false" fast="false">

<listOfReactants>

<speciesReference species="mwdb30ae98_436d_48b0_b344_8c393d0a73fd"/>

</listOfReactants>

<listOfProducts>

<speciesReference species="mw16a20824_e3b6_4f7b_aa76_6a31fe8cbb0c"/>

</listOfProducts>

<kineticLaw>

<math xmlns="http://www.w3.org/1998/Math/MathML">

<apply>

<divide/>

<apply>

<times/>

<ci> mwe7196ae4_92de_4b7f_81eb_664a3190c8c1 </ci>

<ci> mwdb30ae98_436d_48b0_b344_8c393d0a73fd </ci>

</apply>

<apply>

<plus/>

<ci> mw071a335d_d23b_4625_b128_4a852af8a829 </ci>

<ci> mwdb30ae98_436d_48b0_b344_8c393d0a73fd </ci>

</apply>

</apply>

</math>

<listOfParameters>

<parameter id="mwe7196ae4_92de_4b7f_81eb_664a3190c8c1" name="Vm1" value="32" units="MWDERIVEDUNIT_molecule__second"/>

<parameter id="mw071a335d_d23b_4625_b128_4a852af8a829" name="Km1" value="16" units="MWBUILTINUNIT_molecule"/>

</listOfParameters>

</kineticLaw>

</reaction>

<reaction id="mwaf1aaa87_427e_428f_a48c_a48c9398bd05" name="reaction_37" reversible="false" fast="false">

<listOfReactants>

<speciesReference species="mw16a20824_e3b6_4f7b_aa76_6a31fe8cbb0c"/>

</listOfReactants>

<listOfProducts>

<speciesReference species="mwde736097_4b47_42d4_bf63_48c2af8ebf99"/>

</listOfProducts>

<kineticLaw>

<math xmlns="http://www.w3.org/1998/Math/MathML">

<apply>

<divide/>

<apply>

<times/>

<ci> mw3fa63209_f94d_4000_8b8b_67eaa59f6481 </ci>

<ci> mw16a20824_e3b6_4f7b_aa76_6a31fe8cbb0c </ci>

</apply>

<apply>

<plus/>

<ci> mwb63e3a31_e2f0_4c2e_9303_490fc27cc9a9 </ci>

<ci> mw16a20824_e3b6_4f7b_aa76_6a31fe8cbb0c </ci>

</apply>

</apply>

</math>

<listOfParameters>

<parameter id="mw3fa63209_f94d_4000_8b8b_67eaa59f6481" name="Vm1" value="120" units="MWDERIVEDUNIT_molecule__second"/>

<parameter id="mwb63e3a31_e2f0_4c2e_9303_490fc27cc9a9" name="Km1" value="60" units="MWBUILTINUNIT_molecule"/>

</listOfParameters>

</kineticLaw>

</reaction>

<reaction id="mwed1d677b_2ffd_4483_aef2_1f10f3f0fb99" name="reaction_38" reversible="false" fast="false">

<listOfReactants>

<speciesReference species="mwde736097_4b47_42d4_bf63_48c2af8ebf99"/>

</listOfReactants>

<listOfProducts>

<speciesReference species="mw6155c54e_4a7e_4856_a4a9_1fcf68bf7b12"/>

</listOfProducts>

<kineticLaw>

<math xmlns="http://www.w3.org/1998/Math/MathML">

<apply>

<divide/>

<apply>

<times/>

<ci> mw021a7bc7_5ed1_457f_8948_ac53bb989d7d </ci>

<ci> mwde736097_4b47_42d4_bf63_48c2af8ebf99 </ci>

</apply>

<apply>

<plus/>

<ci> mw82fb7137_6e11_4fa7_bb7c_60f9b9e82aa6 </ci>

<ci> mwde736097_4b47_42d4_bf63_48c2af8ebf99 </ci>

</apply>

</apply>

</math>

<listOfParameters>

<parameter id="mw021a7bc7_5ed1_457f_8948_ac53bb989d7d" name="Vm1" value="180" units="MWDERIVEDUNIT_molecule__second"/>

<parameter id="mw82fb7137_6e11_4fa7_bb7c_60f9b9e82aa6" name="Km1" value="90" units="MWBUILTINUNIT_molecule"/>

</listOfParameters>

</kineticLaw>

</reaction>

<reaction id="mw32e3eb72_7833_450a_a040_818afac2f14d" name="reaction_40" reversible="false" fast="false">

<listOfReactants>

<speciesReference species="mw0fc387f1_a053_4273_b301_eee7fd6053ee"/>

</listOfReactants>

<listOfProducts>

<speciesReference species="mwa8bb8232_78cc_4d1b_bc42_a085dfd0941b"/>

</listOfProducts>

<kineticLaw>

<math xmlns="http://www.w3.org/1998/Math/MathML">

<apply>

<times/>

<ci> mw24cc8b60_0117_4c46_b1c0_30e765e2c6b5 </ci>

<ci> mw0fc387f1_a053_4273_b301_eee7fd6053ee </ci>

</apply>

</math>

<listOfParameters>

<parameter id="mw24cc8b60_0117_4c46_b1c0_30e765e2c6b5" name="kForward1" value="0.01" units="MWDERIVEDUNIT_1__second"/>

</listOfParameters>

</kineticLaw>

</reaction>

<reaction id="mw0bff310a_9225_4daf_916a_b521658e98a9" name="reaction_41" reversible="false" fast="false">

<listOfReactants>

<speciesReference species="mwa8bb8232_78cc_4d1b_bc42_a085dfd0941b"/>

</listOfReactants>

<listOfProducts>

<speciesReference species="mwac4fcd3b_4c80_42a8_9264_135e75f1d961"/>

<speciesReference species="mwd994b0ae_580e_4bc9_9dc2_7012c5099e93"/>

</listOfProducts>

<kineticLaw>

<math xmlns="http://www.w3.org/1998/Math/MathML">

<apply>

<divide/>

<apply>

<times/>

<ci> mw125f11dc_5922_47c6_ac63_432235874101 </ci>

<apply>

<power/>

<ci> mwa8bb8232_78cc_4d1b_bc42_a085dfd0941b </ci>

<ci> mw49a37b8a_7a54_4bcd_834d_a852853481d5 </ci>

</apply>

</apply>

<apply>

<plus/>

<ci> mw16e12b75_db77_4bfd_86b5_ec7a76ec2eca </ci>

<apply>

<power/>

<ci> mwa8bb8232_78cc_4d1b_bc42_a085dfd0941b </ci>

<ci> mw49a37b8a_7a54_4bcd_834d_a852853481d5 </ci>

</apply>

</apply>

</apply>

</math>

<listOfParameters>

<parameter id="mw125f11dc_5922_47c6_ac63_432235874101" name="Vm1" value="240" units="MWDERIVEDUNIT_molecule__second"/>

<parameter id="mw49a37b8a_7a54_4bcd_834d_a852853481d5" name="n1" value="1"/>

<parameter id="mw16e12b75_db77_4bfd_86b5_ec7a76ec2eca" name="Kp1" value="1" units="MWBUILTINUNIT_molecule"/>

</listOfParameters>

</kineticLaw>

</reaction>

<reaction id="mwe479b91a_9f7e_41bb_b81b_e94eca7cf2e2" name="reaction_42" reversible="false" fast="false">

<listOfReactants>

<speciesReference species="mw5f4cb81e_f578_43ca_9dbf_6f68315c839e"/>

</listOfReactants>

<listOfProducts>

<speciesReference species="mw725cdfad_dc30_46d0_a778_10f361a8d4f2"/>

</listOfProducts>

<kineticLaw>

<math xmlns="http://www.w3.org/1998/Math/MathML">

<apply>

<times/>

<ci> mw3a33ebdd_5a11_4629_9f6f_624ca4d5dea5 </ci>

<ci> mw5f4cb81e_f578_43ca_9dbf_6f68315c839e </ci>

</apply>

</math>

<listOfParameters>

<parameter id="mw3a33ebdd_5a11_4629_9f6f_624ca4d5dea5" name="kForward1" value="0.01" units="MWDERIVEDUNIT_1__second"/>

</listOfParameters>

</kineticLaw>

</reaction>

<reaction id="mwee9259d4_375f_4cb8_812b_ee3a04b8607e" name="reaction_43" reversible="false" fast="false">

<listOfReactants>

<speciesReference species="mw725cdfad_dc30_46d0_a778_10f361a8d4f2"/>

</listOfReactants>

<listOfProducts>

<speciesReference species="mwea1c0c15_2b67_46fb_929b_7c1267ad9963"/>

</listOfProducts>

<kineticLaw>

<math xmlns="http://www.w3.org/1998/Math/MathML">

<apply>

<times/>

<ci> mw1e244a56_5b02_459d_a902_9a14d022e626 </ci>

<ci> mw725cdfad_dc30_46d0_a778_10f361a8d4f2 </ci>

</apply>

</math>

<listOfParameters>

<parameter id="mw1e244a56_5b02_459d_a902_9a14d022e626" name="kForward1" value="0.083" units="MWDERIVEDUNIT_1__second"/>

</listOfParameters>

</kineticLaw>

</reaction>

<reaction id="mwbd42de7f_46f4_4b43_b13c_c5a909d65a5a" name="reaction_39" reversible="false" fast="false">

<listOfReactants>

<speciesReference species="mw6155c54e_4a7e_4856_a4a9_1fcf68bf7b12"/>

<speciesReference species="mwf3f1e954_4fa7_4d92_b277_2062ac96263a"/>

</listOfReactants>

<listOfProducts>

<speciesReference species="mwf94121d8_c0d7_4448_8774_638eff8c2312"/>

<speciesReference species="mwf3f1e954_4fa7_4d92_b277_2062ac96263a"/>

</listOfProducts>

<kineticLaw>

<math xmlns="http://www.w3.org/1998/Math/MathML">

<apply>

<divide/>

<apply>

<times/>

<ci> mw3f4268ac_93b9_467d_8bf6_c08cb1fd1a22 </ci>

<ci> mw6155c54e_4a7e_4856_a4a9_1fcf68bf7b12 </ci>

</apply>

<apply>

<plus/>

<apply>

<times/>

<ci> mwf6211831_aa47_4433_8800_9c73f8862073 </ci>

<apply>

<plus/>

<cn type="integer"> 1 </cn>

<apply>

<divide/>

<ci> mwf3f1e954_4fa7_4d92_b277_2062ac96263a </ci>

<ci> mw733f4d81_aac5_4c09_bb28_25cfb7ae0c18 </ci>

</apply>

</apply>

</apply>

<ci> mw6155c54e_4a7e_4856_a4a9_1fcf68bf7b12 </ci>

</apply>

</apply>

</math>

<listOfParameters>

<parameter id="mw3f4268ac_93b9_467d_8bf6_c08cb1fd1a22" name="Vm1" value="1000" units="MWDERIVEDUNIT_molecule__second"/>

<parameter id="mwf6211831_aa47_4433_8800_9c73f8862073" name="Km1" value="500" units="MWBUILTINUNIT_molecule"/>

<parameter id="mw733f4d81_aac5_4c09_bb28_25cfb7ae0c18" name="Ki1" value="250" units="MWBUILTINUNIT_molecule"/>

</listOfParameters>

</kineticLaw>

</reaction>

<reaction id="mwb13075ea_350d_4e3e_b96d_3496ea80707c" name="reaction_44" reversible="false" fast="false">

<listOfReactants>

<speciesReference species="mw1c2ae8fe_bef4_408b_a731_b47363bf4c63"/>

</listOfReactants>

<listOfProducts>

<speciesReference species="mw3d1df92d_2bbf_45fa_8039_0f7a0a668b47"/>

</listOfProducts>

<kineticLaw>

<math xmlns="http://www.w3.org/1998/Math/MathML">

<apply>

<times/>

<ci> mw0e85b53d_a5b5_449c_9ac5_c0f26f5b5718 </ci>

<ci> mw1c2ae8fe_bef4_408b_a731_b47363bf4c63 </ci>

</apply>

</math>

<listOfParameters>

<parameter id="mw0e85b53d_a5b5_449c_9ac5_c0f26f5b5718" name="kForward1" value="0.0003050922" units="MWDERIVEDUNIT_1__second"/>

</listOfParameters>

</kineticLaw>

</reaction>

<reaction id="mwc53b183b_f313_4d2a_9d47_ca7db68300ca" name="reaction_45" reversible="false" fast="false">

<listOfReactants>

<speciesReference species="mwca4fe501_c33f_471c_9ec9_b4ef623dd41a"/>

</listOfReactants>

<listOfProducts>

<speciesReference species="mw0fb4af26_aaa9_46cf_820f_a01414045446"/>

</listOfProducts>

<kineticLaw>

<math xmlns="http://www.w3.org/1998/Math/MathML">

<apply>

<times/>

<ci> mw1a4f99b4_2ddd_401c_abe0_f010ed55c2dd </ci>

<ci> mwca4fe501_c33f_471c_9ec9_b4ef623dd41a </ci>

</apply>

</math>

<listOfParameters>

<parameter id="mw1a4f99b4_2ddd_401c_abe0_f010ed55c2dd" name="kForward1" value="1.01e-008" units="MWDERIVEDUNIT_1__second"/>

</listOfParameters>

</kineticLaw>

</reaction>

<reaction id="mwebb93633_2c4a_4d7c_af2e_e5a181936f7a" name="reaction_46" reversible="false" fast="false">

<listOfReactants>

<speciesReference species="mwcbdd87f9_b3ed_4d0a_9e54_0fdbf6fcde76"/>

</listOfReactants>

<listOfProducts>

<speciesReference species="mw37536c65_bf58_4c51_a2ee_d0100642163f"/>

</listOfProducts>

<kineticLaw>

<math xmlns="http://www.w3.org/1998/Math/MathML">

<apply>

<times/>

<ci> mw6a1cc12f_56d2_4d0b_a93f_c844e3301039 </ci>

<ci> mwcbdd87f9_b3ed_4d0a_9e54_0fdbf6fcde76 </ci>

</apply>

</math>

<listOfParameters>

<parameter id="mw6a1cc12f_56d2_4d0b_a93f_c844e3301039" name="kForward1" value="1.01e-009" units="MWDERIVEDUNIT_1__second"/>

</listOfParameters>

</kineticLaw>

</reaction>

<reaction id="mw9df7006f_97a1_4e16_a8ed_b373e1ed6d07" name="reaction_13" reversible="false" fast="false">

<listOfReactants>

<speciesReference species="mwa5ec613a_4816_4904_9b5e_7e9b01695b91"/>

</listOfReactants>

<listOfProducts>

<speciesReference species="mw0e810310_40ad_4cfe_ad6f_889093f29968"/>

<speciesReference species="mw36823663_83dc_47b1_ade9_865411917d6d"/>

</listOfProducts>

<kineticLaw>

<math xmlns="http://www.w3.org/1998/Math/MathML">

<apply>

<divide/>

<apply>

<times/>

<ci> mwac67a136_75e5_4ad8_92f8_cd384c3ec2ae </ci>

<apply>

<power/>

<ci> mwa5ec613a_4816_4904_9b5e_7e9b01695b91 </ci>

<ci> mwe06d83c9_1f0e_4f06_9b63_8c20fb7c5c9d </ci>

</apply>

</apply>

<apply>

<plus/>

<ci> mwe1734793_4320_44ed_a72e_ed4bb5fdd4e2 </ci>

<apply>

<power/>

<ci> mwa5ec613a_4816_4904_9b5e_7e9b01695b91 </ci>

<ci> mwe06d83c9_1f0e_4f06_9b63_8c20fb7c5c9d </ci>

</apply>

</apply>

</apply>

</math>

<listOfParameters>

<parameter id="mwac67a136_75e5_4ad8_92f8_cd384c3ec2ae" name="Vm1" value="70" units="MWDERIVEDUNIT_molecule__second"/>

<parameter id="mwe1734793_4320_44ed_a72e_ed4bb5fdd4e2" name="Kp1" value="1" units="MWBUILTINUNIT_molecule"/>

<parameter id="mwe06d83c9_1f0e_4f06_9b63_8c20fb7c5c9d" name="n1" value="1"/>

</listOfParameters>

</kineticLaw>

</reaction>

<reaction id="mwe55b8dba_dda6_4abe_9c4f_f6f8e7f463ce" name="reaction_15" reversible="false" fast="false">

<listOfReactants>

<speciesReference species="mw36823663_83dc_47b1_ade9_865411917d6d"/>

</listOfReactants>

<listOfProducts>

<speciesReference species="mweb909918_2d84_489e_a6e4_a8b51c6b2040"/>

</listOfProducts>

<kineticLaw>

<math xmlns="http://www.w3.org/1998/Math/MathML">

<apply>

<times/>

<ci> mw4d300372_cc99_48f6_a87a_e90d758b9744 </ci>

<ci> mw36823663_83dc_47b1_ade9_865411917d6d </ci>

</apply>

</math>

<listOfParameters>

<parameter id="mw4d300372_cc99_48f6_a87a_e90d758b9744" name="kForward1" value="0.02" units="MWDERIVEDUNIT_1__second"/>

</listOfParameters>

</kineticLaw>

</reaction>

</listOfReactions>

</model>

</sbml>
